# Engineering pH‐Responsive Nanocarriers via an Optimized Synthesis of PMOXA‐*b*‐PDPA Amphiphilic Diblock Copolymers

**DOI:** 10.1002/marc.202500418

**Published:** 2025-07-28

**Authors:** John Peter Coats, Anamarija Nikoletić, Lukas Heuberger, Voichita Mihali, Cora‐Ann Schoenenberger, Ionel Adrian Dinu, Cornelia G. Palivan

**Affiliations:** ^1^ Department of Chemistry University of Basel Basel Switzerland; ^2^ Swiss Nanoscience Institute University of Basel Basel Switzerland

**Keywords:** cargo encapsulation, polymer synthesis, self‐assembly, triggered release

## Abstract

pH‐responsive nanocarriers have gain significant attention due to their ability to provide controlled cargo delivery with high precision in response to specific stimuli. However, the polymers used in the self‐assembly of these nanocarriers must be carefully designed to meet the requirements of bio‐relevant delivery. Here, we present an optimized synthesis of poly(2‐methyl‐2‐oxazoline)‐*block*‐poly(2‐(diisopropylamino)ethyl methacrylate) (PMOXA‐*b*‐PDPA) block copolymers tailored for obtaining carriers with vesicle architecture and thin membranes for an improved release behavior. By systematically modifying the synthesis conditions, we obtain a small library of copolymers, focusing on low molecular weight (MW) variants to reduce the membrane thickness of the resulting vesicles. We investigate the impact of membrane thickness on the kinetics and efficiency of cargo release in response to a pH shift from neutral to slightly acidic conditions that are particularly relevant in pathological environments like tumors. Model cargos of varying MWs, including doxorubicin hydrochloride, exhibited differential release profiles under these pH conditions. Together with no cytotoxicity, the thin membrane represents key aspects that support further development of such carriers for therapeutic applications.

## Graphical abstract

pH‐responsive nanocarriers are gaining attention for controlled cargo delivery in response to specific stimuli. The synthesis of poly(2‐methyl‐2‐oxazoline)‐*block*‐poly(2‐(diisopropylamino)ethyl methacrylate) (PMOXA‐*b*‐PDPA) copolymers is optimized to create nano‐ and micrometer sizes vesicles serving as carriers with thin membranes. The nanocarriers show improved cargo release in response to mild pH shifts analogous to those seen in tumor environments, with no cytotoxicity, making them a promising platform for therapeutic applications.

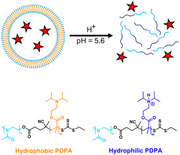

## 1 Introduction

Amphiphilic block copolymers (BCPs) are increasingly studied as key components in the bottom‐up design of biocompatible carriers, each offering distinct properties and functions [1, 2, 3] for applications ranging from drug delivery to nanoreactors and cell‐mimics [4, 5, 6, 7, 8, 9]. Compared to traditional lipid‐based nanocarriers, the ones consisting of BCPs offer several advantages, such as enhanced chemical and physical stability, reduced membrane permeability, and greater versatility for chemical modifications. These features enable the customization of properties and functionalities to suit particular needs.

Of particular interest are polymeric nanocarriers that address key challenges in pharmacology, including delivery of poorly soluble drugs and imaging agents with short circulation times [10], to specific areas of the body [11, 12]. Due to the complex scenario of in vivo conditions the nanocarriers have to be optimized in respect to several properties in order to reach the desired bio‐location and not lose the cargo in undesired regions, promote “on demand” delivery, and being biocompatible. For example, various surface modifications of nanocarriers have been reported to optimize the delivery, such as PEGylation for extended retention [13], incorporation of cell‐penetrating peptides to enhance cellular uptake [14], and organic chemistry alterations for the addition of new functional groups, often compatible with click chemistry [15, 16]. Another strategy to advance carriers is to use stimuli‐responsive BCPs, as they ensure delivery at sites where specific stimuli (e.g., pH, redox, or chemical) are present or in response to externally applied triggers (e.g., light, magnetism, or sonication) [17]. Among these, pH‐responsive polymers result in nanocarriers which are promising for targeting acidic tumor microenvironments [18] where the pH is significantly lower (5.6–6.8) compared to that of healthy tissues (7.4) [19]. This difference, caused by factors such as glycolysis, hypoxia, and poor blood perfusion [20], makes polyacids and polybases with a pK_a_ between 6.0 and 7.0 ideal candidates for cancer‐targeted delivery. Upon protonation in the acidic environment of the tumor, these polymers undergo structural and hydrophilic/hydrophobic transitions, leading to controlled cargo release or accumulation of imaging agents at the target site [18]. While specific properties of the carriers (e.g. membrane thickness of vesicles) are essential to support efficient delivery by controlling the necessary time for release upon the presence of the stimulus, they are underexplored in general synthetic routes of BCPs. The real question when an efficient delivery is aimed, is how to optimize/change a synthetic route to obtain BCPs inducing carriers with fine‐tuned properties. Tuning the properties imposes adaptations/changes of the synthesis route, without which the delivery is limited or has no space and time precision.

In this study, we present an optimized synthesis of poly(2‐methyl‐2‐oxazoline)‐*block*‐poly[2‐(diisopropylamino)ethyl methacrylate] (PMOXA‐*b*‐PDPA) diblock copolymers and end‐group coupling reactions aiming to generate carriers with thin membranes supporting stimuli‐driven cargo delivery. An essential aspect was to obtain carriers with vesicle architecture, sizes ranging from nano‐ to micrometer, reduced membrane thickness for an efficient cargo release, and even specifically functionalized for further targeting approaches. We selected PMOXA as one of the blocks because it is known as biocompatible and protein repellent [21]. PDPA, with a pK_a_ of 6.8, was selected as an ideal hydrophobic polymer candidate for stimuli‐responsive applications, as it allows for >80% protonation of its tertiary amine groups at pH 5.5 and 37°C [22], causing a significant change in solubility that enhances drug release in acidic conditions [23]. We modified the synthesis conditions and replaced the commercially available dithiobenzoate chain transfer agent (CTA) with an in‐house synthesized trithiocarbonate (TTC). We were interested to eliminate the issues related to dithiobenzoate hydrolysis and enable the synthesis of lower molecular weight (MW) polymers via photoiniferter reversible addition‐fragmentation chain‐transfer (PI‐RAFT) polymerization. In addition, we introduced a tailorable end‐group on the hydrophilic block to achieve a versatile “clickable” site, supporting further development of these carriers for targeting applications or immobilization on surfaces. One major challenge with current PDPA‐based self‐assemblies is the uncontrolled cargo leakage over time, which occurs regardless of pH where acidic conditions only increase the release kinetics [23]. In this study, we encapsulate a range of model cargos, assess their stability within the carrier, and examine their pH‐responsive release behavior. Our results show that under physiological conditions (pH 7.4), less than 20% of the cargo is released, while acidification to tumor‐like conditions (as low as pH 5.6) [18] triggers the release of up to 99% of the cargo. To better understand what influences release efficiency, we investigated the interplay between copolymer MW and cargo chemistry. We also assessed the cytotoxicity of the self‐assembled diblock copolymer systems in HeLa cells which is an essential step toward their further biomedical application. Optimization of the synthesis and self‐assembly processes for PMOXA‐*b*‐PDPA copolymers was key to developing carriers capable of controlled, pH‐triggered cargo release.

## 2 Results and Discussion

### 2.1 Synthesis of Prop‐PMOXA_n_‐*b*‐PDPA_m_

To produce amphiphilic diblock copolymers able to form self‐assembled compartments with pH‐sensitivity, a hydrophobic block containing bulky, protonable tertiary amine moieties as side chains was selected. This design enables precise tuning of block copolymer amphiphilicity in response to environmental pH changes. In addition, we functionalized the copolymers by the presence of a propargyl (referred to as prop‐) functionality as a tailorable end‐group on the hydrophilic block to introduce a versatile “clickable” site. This functionalization facilitates the generation of nanocarriers with various functional units on their surface after the self‐assembly.

**FIGURE 1:**
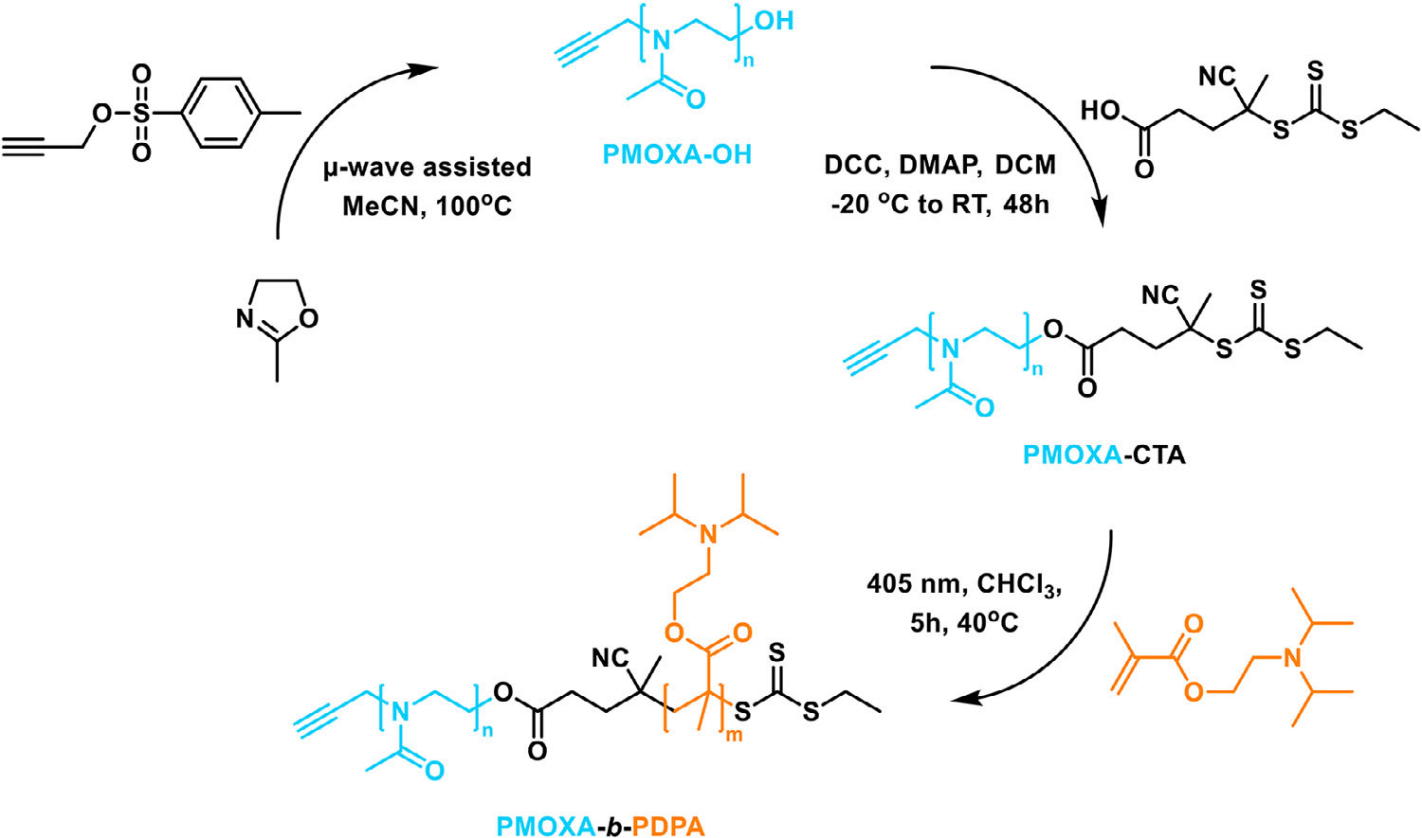
Reaction scheme for the synthesis of poly(2‐methyl‐2‐oxazoline)‐*b*‐poly[2‐(diisopropylamino)ethyl methacrylate] (PMOXA_n_‐*b*‐PDPA_m_).

The synthesis of a small library of low MW prop‐PMOXA_n_‐*b*‐PDPA_m_ was achieved by a three‐step process (Figure 1): (i) cationic ring‐opening polymerization (CROP) of 2‐methyl‐2‐oxazoline (MOXA) using a propargyl p‐toluenesulfonate (prop‐TOS) initiator to produce the alkyne terminated prop‐PMOXA_n_‐OH homopolymer; (ii) esterification of prop‐PMOXA_n_‐OH with a carboxylic acid‐functionalized CTA to form the macro‐CTA, and (iii) chain extension of prop‐PMOXA_n_‐CTA with 2‐(diisopropylamino)ethyl methacrylate (DPA) via photoiniferter reversible addition–fragmentation chain transfer (PI‐RAFT) polymerization to yield the diblock copolymers with desired pH‐responsive block lengths (Figure 1). The values of “n” and “m” were controlled by adjusting the monomer‐to‐initiator molar ratio in the polymerization step. MOXA and prop‐TOS ratio determined the length of the PMOXA block, while the subsequent polymerization of DPA under PI‐RAFT conditions enabled precise tuning of the PDPA block length.

**FIGURE 2:**
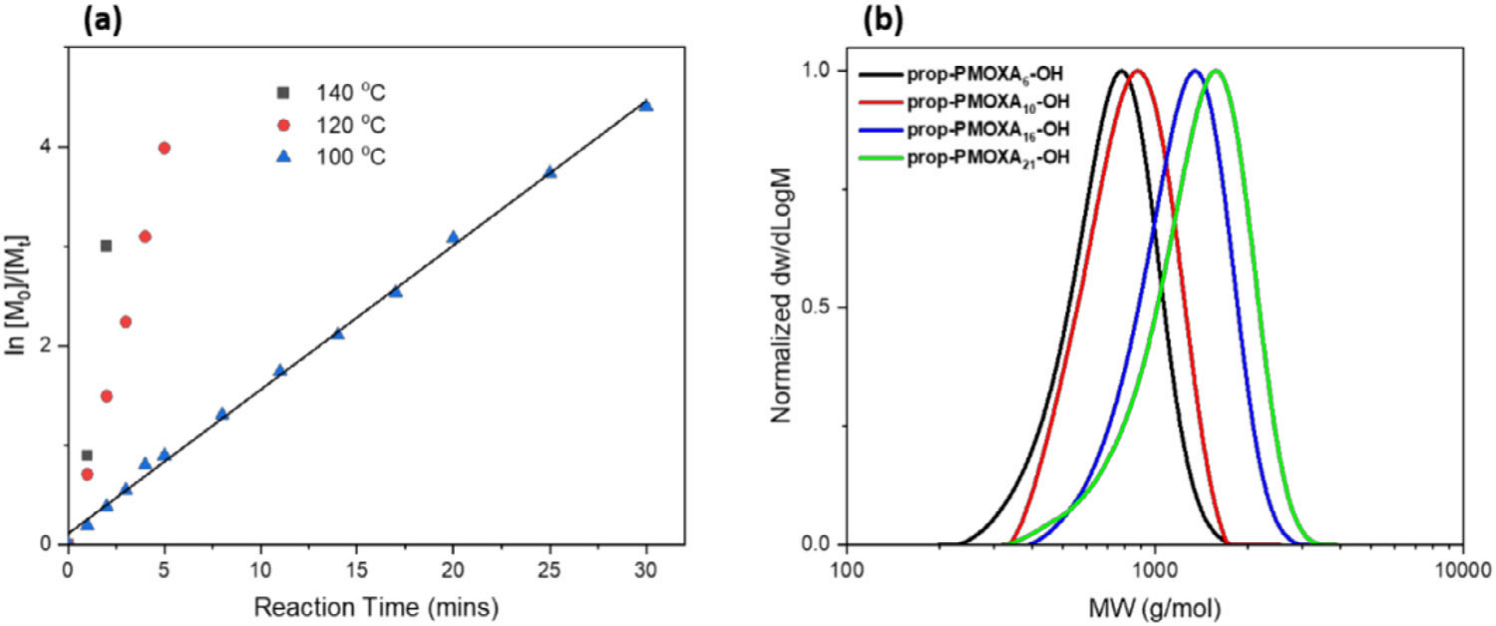
(a) Kinetics of MOXA monomer conversion over time, evaluated by ^1^H NMR spectroscopy, demonstrating the pseudo‐first‐order reaction process at 100°C for 20:1 monomer‐to‐initiator molar ratio. (b) GPC profiles of all prop‐PMOXA_n_‐OH homopolymers.

In the first step, microwave‐assisted CROP of prop‐PMOXA_n_‐OH was optimized by adjusting the reaction parameters from a previously established reaction to obtain low MW polymers [24, 25]. Specifically, the reaction temperature was reduced from 140°C to 100°C and the reaction time was extended to 30 min. These reaction parameter modifications were necessary for accurate quenching of the polymerization such to precisely target shorter block lengths with minimal side reactions. The slower rate of the homopolymerization at reduced temperature improved monomer conversion and ensured first‐order reaction kinetics, providing excellent control over the MW and dispersity (*Ð* = 1.06–1.10). The polymerization reaction kinetics of prop‐PMOXA_n_‐OH were evaluated by ^1^H NMR spectroscopy (Figure 2a). The analysis indicates that the cationic polymerization maintains a perfectly linear growth of polymer chains (adj. R^2^ = 0.998) throughout the reaction up to full monomer conversion without detectable side reactions of chain transfer. The pseudo‐first‐order kinetics were determined by the linear growth of ln[M_0_]/[M_t_] against time, where the linear correlation is calculated with the adjusted R^2^ model. Each prop‐PMOXA_n_‐OH homopolymer was then analyzed by GPC to determine the MW dispersity and all polymers showed monomodal weight distributions (Figure 2b).

In the second step, the TTC RAFT agent was conjugated to the prop‐PMOXA_n_‐OH homopolymer via Steglich esterification using *N,N’*‐dicyclohexylcarbodiimide (DCC) and 4‐(dimethylamino)pyridine (DMAP) to activate the TTC carboxylic acid (Figure 1). The TTC was selected due to its increased hydrolytic stability compared to the more common dithiobenzoate RAFT agents, which are prone to hydrolysis in the presence of the highly hygroscopic PMOXA homopolymers. The higher stability of PMOXA‐TTC macroinitiators prevents the issues of macroinitiator degradation and uncontrolled termination during subsequent polymerizations. TTC also grants the potential use of PI‐RAFT processes, eliminating the need for sacrificial radical‐forming species to initiate the polymerization, thereby preventing undesired homopolymer formation.

In the third step, the prop‐PMOXA_n_‐CTA was chain extended via PI‐RAFT polymerization to form the final diblock copolymers under optimized conditions (Figure 1). The degassing process of the PI‐RAFT reaction mixture prior to polymerization was simplified by minimizing the reaction vessel headspace, reducing the required nitrogen purging time to just 5–10 min [26]. This allows for a far less rigorous deoxygenation, simplifying both polymerization setup and workup. These improvements allowed the products to be easily tailored and reproduced.

**FIGURE 3:**
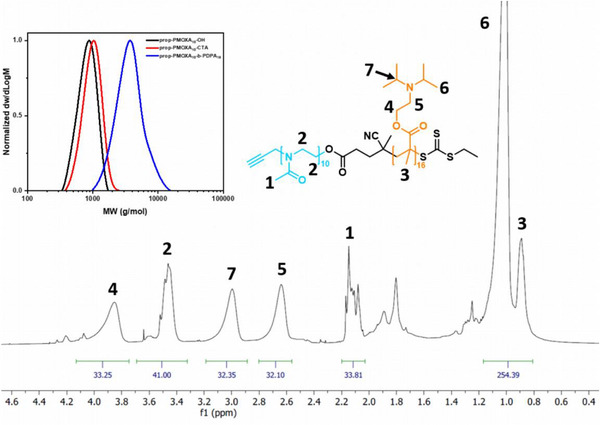
Structural characterization of prop‐PMOXA_10_‐*b*‐PDPA_16_ by GPC and ^1^H NMR (500 MHz, CDCl_3_).

PI‐RAFT polymerization kinetics were monitored by ^1^H NMR spectroscopy and indicated full monomer conversion within 5 h at 40°C and under constant UV irradiation. All diblocks were purified by dialysis using low MWCO (1 kDa) regenerated cellulose membranes, which effectively removed unreacted monomer and unfunctionalized prop‐PMOXA_n_‐OH homopolymer. The diblock copolymers were characterized by ^1^H NMR spectroscopy, GPC, and DSC analysis and compared with the corresponding PMOXA‐TTC macroinitiators, confirming their tailored MWs, narrow MW dispersity, and high purity (Figure 3 and Table 1 and Figures S4–S9).

**TABLE 1:** Characterization data of prop‐PMOXA_n_‐*b*‐PDPA_m_ diblock copolymers.

Composition	M_n_ (Da)^a^	Ð^b^	*f*‐ratio^c^
PMOXA_6_‐*b*‐PDPA_11_	3100	1.17	18
PMOXA_10_‐*b*‐PDPA_16_	4500	1.19	20
PMOXA_16_‐*b*‐PDPA_30_	8000	1.18	18
PMOXA_21_‐*b*‐PDPA_34_	9300	1.20	20
PMOXA_16_‐*b*‐PDPA_42_	10 600	1.19	13

^a^ Calculated from ^1^H NMR spectroscopy.

^b^ Obtained by GPC.

^c^ Calculated using the equation *f* = M_n_(PMOXA) / (M_n_(PMOXA) + M_n_(PDPA)).

Interestingly, efficient polymerization was achieved under 405 nm irradiation without the addition of a photo‐initiator, despite previous reports indicating low reactivity of similar trithiocarbonyl‐based CTAs under these conditions [27]. Unlike aqueous PISA systems, where phase separation may limit monomer and radical mobility, our polymerization was carried out in chloroform, a homogeneous and non‐selective solvent in which both PMOXA and PDPA blocks remain fully soluble. This uniform environment facilitates efficient diffusion and RAFT equilibrium. Additionally, the reaction temperature of 40°C likely favors the homolytic cleavage of the CTA, enhancing radical generation despite its weak n → *π* ^⁎^ absorption at 405 nm. These combined effects, solvent environment, thermal assistance, and homogeneous reaction conditions, contribute to the improved polymerization efficiency observed in our system.

### 2.2 Prop‐PMOXA_n_‐*b*‐PDPA_m_ End‐Group Functionalization

To further enhance the properties of the copolymers, we introduced a propargyl‐containing initiator during the PMOXA synthesis, resulting in diblock copolymers with terminal alkyne groups, prop‐PMOXA_n_‐*b*‐PDPA_m_. These propargyl‐functionalized end‐groups allow for precise modifications through the well‐established copper‐catalyzed azide‐alkyne cycloaddition (CuAAC) click chemistry, providing the flexibility to tailor the copolymers for specific applications. We demonstrated the tunable functionality of prop‐PMOXA_n_‐*b*‐PDPA_m_ using a higher MW analogue, prop‐PMOXA_16_‐*b*‐PDPA_42_ (MW = 10.4 kDa). We hypothesize that the increased MW of this copolymer leads to a more sterically hindered terminal alkyne group, which slows the reaction kinetics and limits its overall functionality. Therefore, the coupling efficiency observed with the higher MW polymers serves as a benchmark that lower MW polymers should meet or exceed.

**FIGURE 4:**
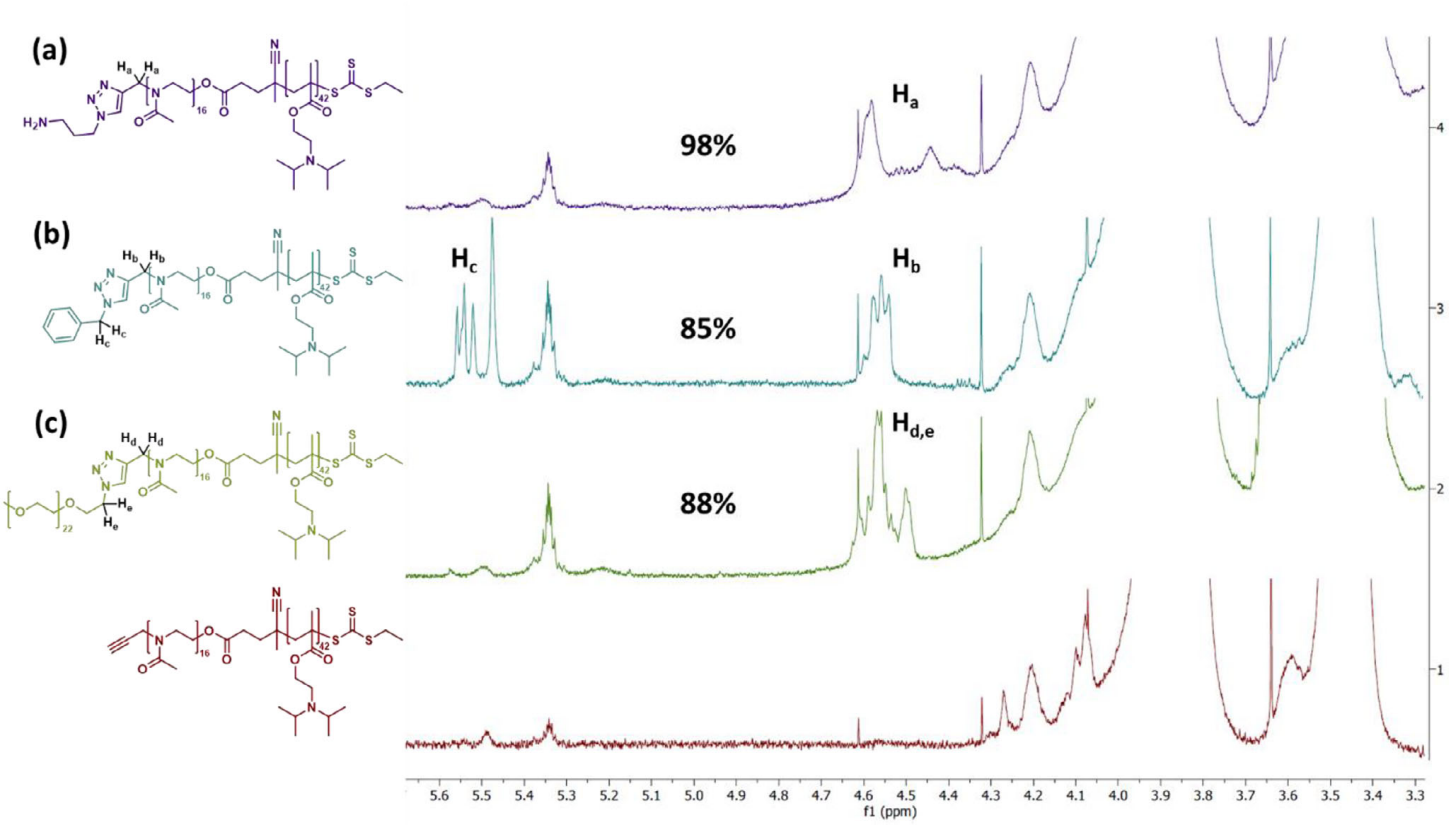
Quantification by ^1^H NMR spectroscopy of the functionalization efficiency after prop‐PMOXA_16_ *‐b‐*PDPA_42_ click reactions with (a) 3‐azido‐1‐propanamine, (b) benzyl azide, and (c) PEG_22_‐azide.

The click reaction was performed under inert nitrogen atmosphere using Cu(I)Br stabilized with PMDETA in DMF to solubilize all components. This approach creates a more versatile reaction setup where a large variety of clickable reagents can be coupled to the copolymer. This versatility was demonstrated by coupling three different reagents to the prop‐PMOXA‐*b*‐PDPA copolymer. Specifically, an aliphatic and an aromatic small azide molecule, and the N_3_‐PEG_22_ homopolymer. After purification by dialysis (MWCO 1 kDa), the products were analyzed by ^1^H NMR spectroscopy to quantify the degree of functionalization (Figure 4). The 3‐azido‐1‐propanamine produced the highest degree of functionalization (98%), followed by the N_3_‐PEG_22_ homopolymer (88%) and the benzyl azide (85%) (Figure 4). These stark differences in polarity, aromaticity, and MW demonstrate the chemical variety that is compatible with this coupling reaction. This approach enables the creation of self‐assembling polymers with highly tailorable end‐group functionalities on the hydrophilic block. The positioning of these moieties as polymer chain end groups ensures their accessibility on the surfaces of nanocarriers after self‐assembly, facilitating their precise tailoring for various applications.

### 2.3 Supramolecular Self‐Assembly of Prop‐PMOXA_n_‐*b*‐PDPA_m_

**FIGURE 5:**
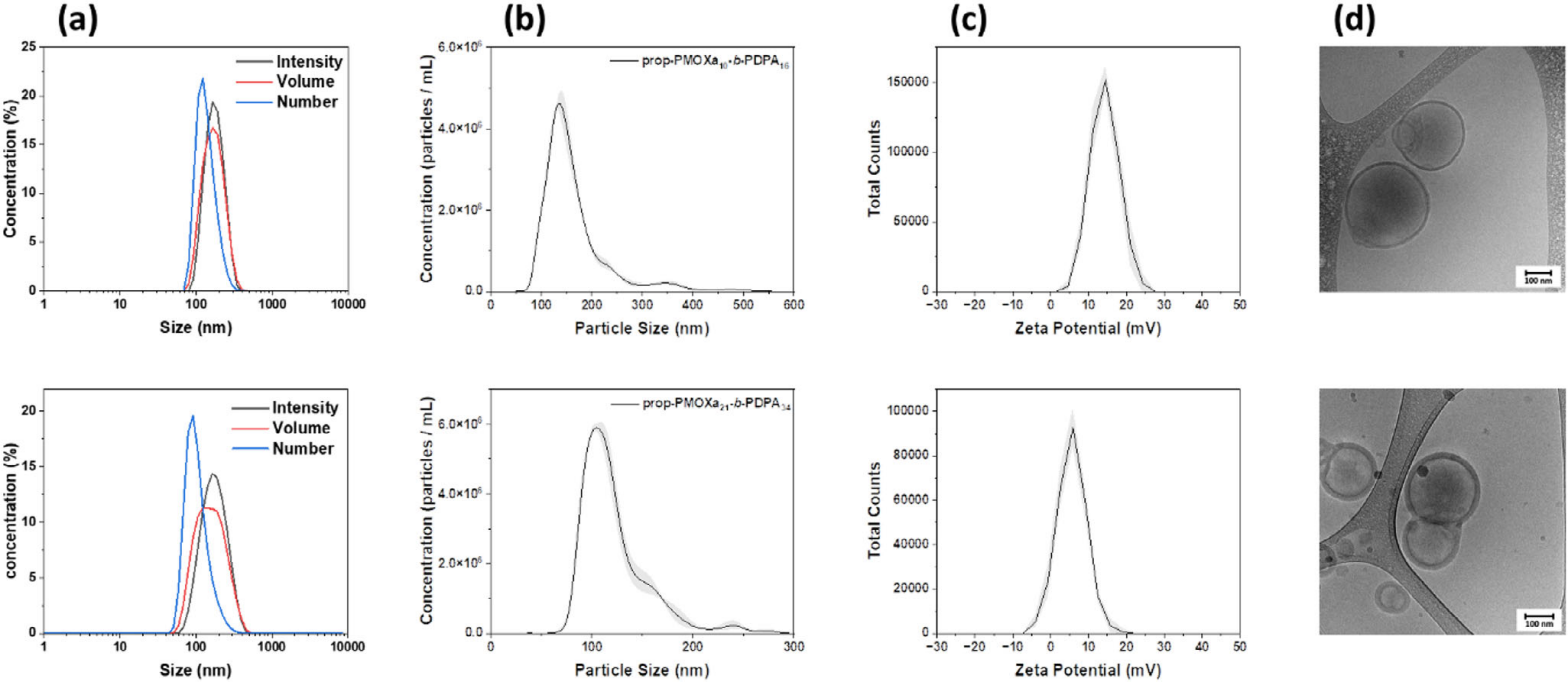
Characterization of the supramolecular assemblies of prop‐PMOXA_10_‐*b*‐PDPA_16_ (top) and prop‐PMOXA_21_‐*b*‐PDPA_34_ (bottom) by (a) DLS, (b) NTA, (c) zeta potential, and (d) cryo‐TEM.

To prepare pH‐responsive polymer nanocarriers, all four copolymers were self‐assembled using the solvent switch method [2], followed by SEC to remove polymer aggregates. The purified self‐assemblies were characterized by a combination of dynamic light scattering (DLS) to determine their hydrodynamic diameters (Figure 5a and Figure S10), nanoparticle tracking analysis (NTA) to assess particle concentration (Figure 5b and Figure S11), zeta potential to investigate the colloidal stability of the nanoassemblies (Figure 5c), and cryo‐transmission electron microscopy (cryo‐TEM) to examine their morphology and membrane thickness (Figure 5d; Figures S12 and S13 and Table S1). The resulting supramolecular self‐assemblies were in the appropriate size range (100–300 nm) for vesicle architecture without the presence of micelles or large aggregate impurities. Additionally, the zeta potential measurements of prop‐PMOXA_10_‐*b*‐PDPA_16_ and prop‐PMOXA_21_‐*b*‐PDPA_34_ assemblies (Figure 5c) showed that they have a slightly positive surface charge. The vesicles were analyzed using cryo‐TEM (Figure 5d), indicating membrane thickness variations between different polymer vesicles. Thus, prop‐PMOXA_6_‐*b*‐PDPA_11_ vesicles exhibited a membrane thickness of 10.5 ± 1.4 nm, while prop‐PMOXA_10_‐*b*‐PDPA_16_ and prop‐PMOXA_16_‐*b*‐PDPA_30_ had increased thicknesses of 13.8 ± 2.8 nm and 14.2 ± 2.0 nm, respectively. The thickest membranes were observed for prop‐PMOXA_21_‐b‐PDPA_34_, with a value of 16.7 ± 1.8 nm, its increase in hydrophobic block length (Table S1 and Figures S12 and S13). Although NTA measurements indicate that all prop‐PMOXA_n_‐*b*‐PDPA_m_ have similar sizes (Figure 5b and Table S1), DLS and cryo‐TEM micrographs show that the vesicles formed by prop‐PMOXA_6_‐*b*‐PDPA_11_, which have the thinnest membranes, appear larger in diameter compared to the other vesicle structures (Figure 5d; Figures S12 and S13). The observed trend in cryo‐TEM size, i.e. larger vesicles for thinner membranes (prop‐PMOXA_6_‐*b*‐PDPA_11_) and smaller vesicles for thicker membranes (prop‐PMOXA_21_‐*b*‐PDPA_34_), is consistent with established physical models for polymer vesicles, where the bending rigidity of diblock copolymer membranes scales quadratically with membrane thickness [28]. As a result, vesicles with thinner, more flexible membranes favor larger radii of curvature, while those with thicker, stiffer membranes adopt smaller sizes.

The differences in membrane thickness between these copolymers are significant, thus reinforcing the need for a highly controlled synthesis when a specific thickness is desired for applications. Our approach enabled the synthesis of low MW diblocks, leading to vesicles with a membrane thickness that is well‐suited for efficient cargo delivery.

### 2.4 Cargo Encapsulation in Prop‐PMOXA_n_‐*b*‐PDPA_m_ Vesicles and pH‐Triggered Release

To see whether the copolymers tolerate formation of micrometer‐sized vesicles allowing to enlarge the applicability of such copolymers, we used double emulsion microfluidics as this offers improved reproducibility, size uniformity, and significant encapsulation efficiency [7]. The double emulsion used PBS (pH 7.4) as the inner and outer aqueous phase. We successfully generated giant unilamellar vesicles (GUVs) based on prop‐PMOXA_10_‐*b*‐PDPA_16_ copolymer. It is known that GUVs are intrinsically less stable than nano‐sized vesicles, resulting in greater permeability due to looser packing, greater chain mobility, and more uniform membrane thickness [29]. Consequently, GUVs can be used to evaluate the membrane permeability because if the cargo cannot pass through the membrane of GUVs, this will not escape from the inner cavity, and thus it should also be suitable for encapsulation in nano‐sized vesicles.

To determine membrane permeability, empty GUVs were mixed with fluorescent dyes of increasing MW for 1 h. Dye permeation into the GUVs was tracked using CLSM, which allows visualization of an increase in the fluorescence intensity inside GUVs upon penetration of the dyes (Figures S14 and S15). The polymer GUV membranes were permeable to resorufin (MW = 213 Da) and sulforhodamine B (MW = 559 Da), whereas no significant permeation was found for carboxyfluorescein (MW = 376 Da), calcein (MW = 622 Da), or ATTO 488 (MW = 804 Da) as indicated by the very low fluorescent signal inside the cavity of GUVs (Figure S14). Therefore, our vesicles (GUVs and consequently nano‐sized vesicles) are able to keep a variety of different cargos inside their cavity without uncontrolled release, while some smaller cargos are released over time.

**FIGURE 6:**
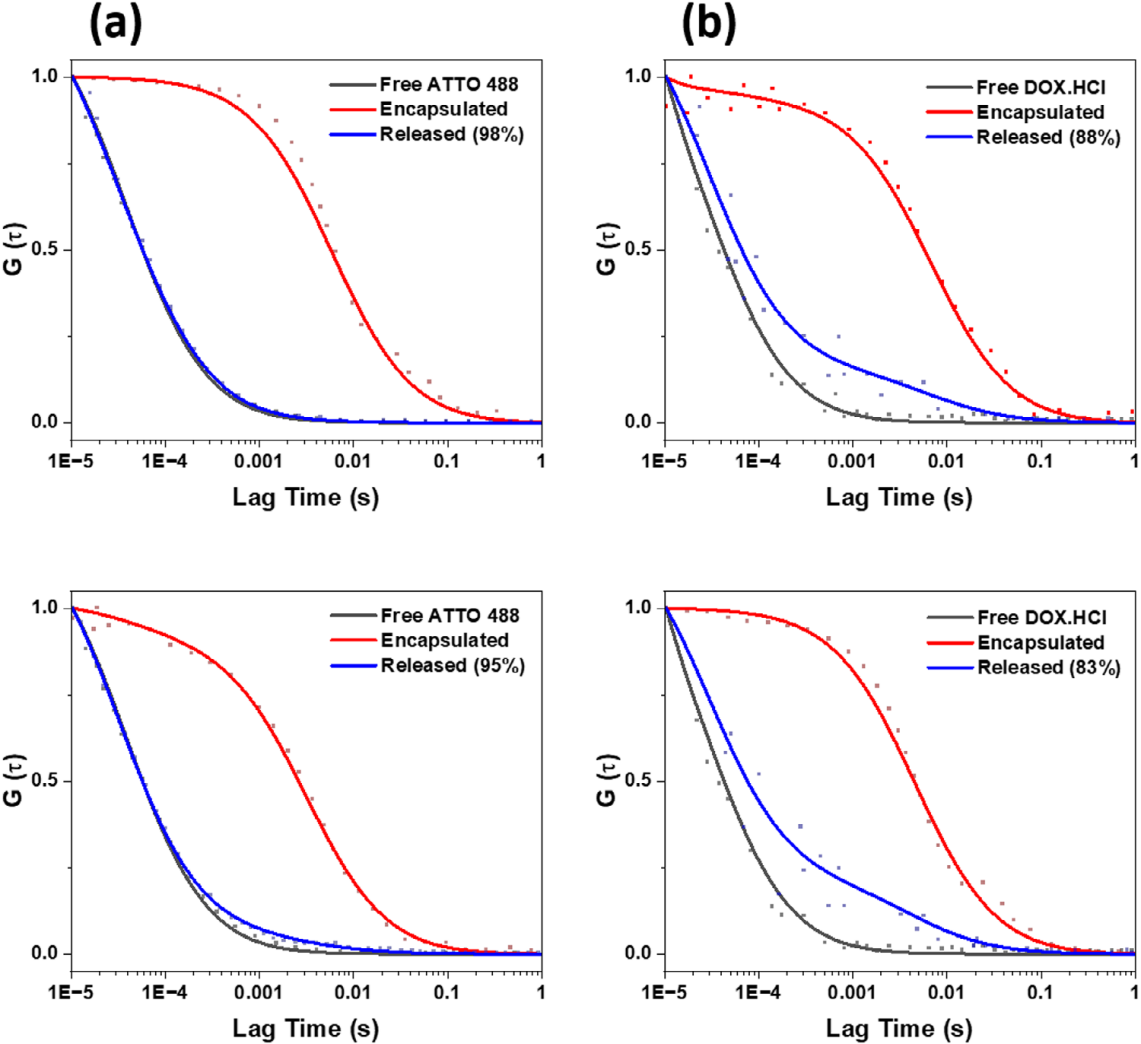
Normalized FCS autocorrelation curves (dots) and corresponding fit curves (lines) of vesicles self‐assembled from (top) prop‐PMOXA_10_‐*b*‐PDPA_16_ and (bottom) prop‐PMOXA_21_‐*b*‐PDPA_34_ copolymers and containing cargo molecules and their pH‐triggered release: (a) ATTO 488 and (b) DOX∙HCl. The data represent the mean of 20 independent measurements.

Then, we used nano‐sized vesicles to evaluate the cargo delivery, as they are expected to support cellular uptake. ATTO 488 or doxorubicin hydrochloride (DOX∙HCl) (MW = 580 Da) were encapsulated in the cavity of PMOXA_n_‐*b*‐PDPA_m_ nano‐sized vesicles during the self‐assembly process. ATTO 488 was used as a model molecule to evaluate the release behavior, while DOX∙HCl, a gold standard anti‐cancer chemotherapy drug, was selected to assess the release efficiency of a therapeutically relevant compound. The fluorescence of both molecules simplifies the release quantification measurements by avoiding chromatographic methods. The encapsulation and release of ATTO 488 and DOX∙HCl were monitored using fluorescence correlation spectroscopy (FCS) (Figure 6). First, the diffusion time of free ATTO 488 and DOX∙HCl in solution was calculated (Equation 1). Then, the diffusion time of encapsulated ATTO 488 and DOX∙HCl, respectively, was determined by a two‐component diffusion fit of the autocorrelation curves based on the sum between a fraction of free diffusing cargos and a fraction of encapsulated ones inside the vesicles (Equation 3). This approach was also used to determine if any fraction of the cargo remained unencapsulated in the solution after the purification process. Finally, we determined the percentage release of cargo after 24 h for ATTO 488 and 48 h for DOX∙HCl. For ATTO 488‐loaded vesicles, its encapsulation was confirmed by the significant increase in diffusion time (τ_D_) from 39 ± 3.8 µs for free ATTO 488 to 5956 ± 2419 µs for ATTO 488‐loaded prop‐PMOXA_10_‐*b*‐PDPA_16_ vesicles and to 3166 ± 1479 µs for ATTO 488‐loaded prop‐PMOXA_21_‐b‐PDPA_34_ vesicles, respectively. The marked increase in fluorophore diffusion time indicates the successful encapsulation of the dye in both types of vesicles (Figure 6a and Tables S2 and S3). After triggering cargo release by adjusting the pH to 5.6 and equilibrating for 24 h, the diffusion times obtained indicated significant dye release. Release efficiencies of 98% and 95% were determined for vesicles of prop‐PMOXA_10_‐*b*‐PDPA_16_ and prop‐PMOXA_21_‐b‐PDPA_34_, respectively. Notably, the prop‐PMOXA_10_‐*b*‐PDPA_16_ vesicles having a thinner membrane (Figure 5d) consistently showed a slightly higher release efficiency (Figure 6a). A similar trend was observed for DOX∙HCl‐loaded vesicles: after lowering the pH to 5.6 and equilibration for 48 h, the measured diffusion times indicated significant release, with efficiencies of 88% for prop‐PMOXA_10_‐*b*‐PDPA_16_ vesicles and 83% for prop‐PMOXA_21_‐*b*‐PDPA_34_ vesicles, respectively (Figure 6b). The release efficiency for DOX∙HCl was lower than for ATTO 488, likely due to the tendency of DOX∙HCl to cluster and crystalize in the aqueous medium where the degree of clustering is correlated with the local concentration [30]. In addition, the consistently reduced release efficiency of prop‐PMOXA_21_‐*b*‐PDPA_34_ vesicles compared to prop‐PMOXA_10_‐*b*‐PDPA_16_ vesicles emphasizes the need for a well‐controlled block copolymer synthesis to minimize its MW and decrease the membrane thickness of the resulting vesicles. We then calculated the hydrodynamic radius (*R*_h_) of the vesicles (Equation 5, Tables S2 and S3). The values of *R*_h_ are in good agreement with the DLS and NTA data for the unloaded vesicles (Table S1).

**FIGURE 7:**
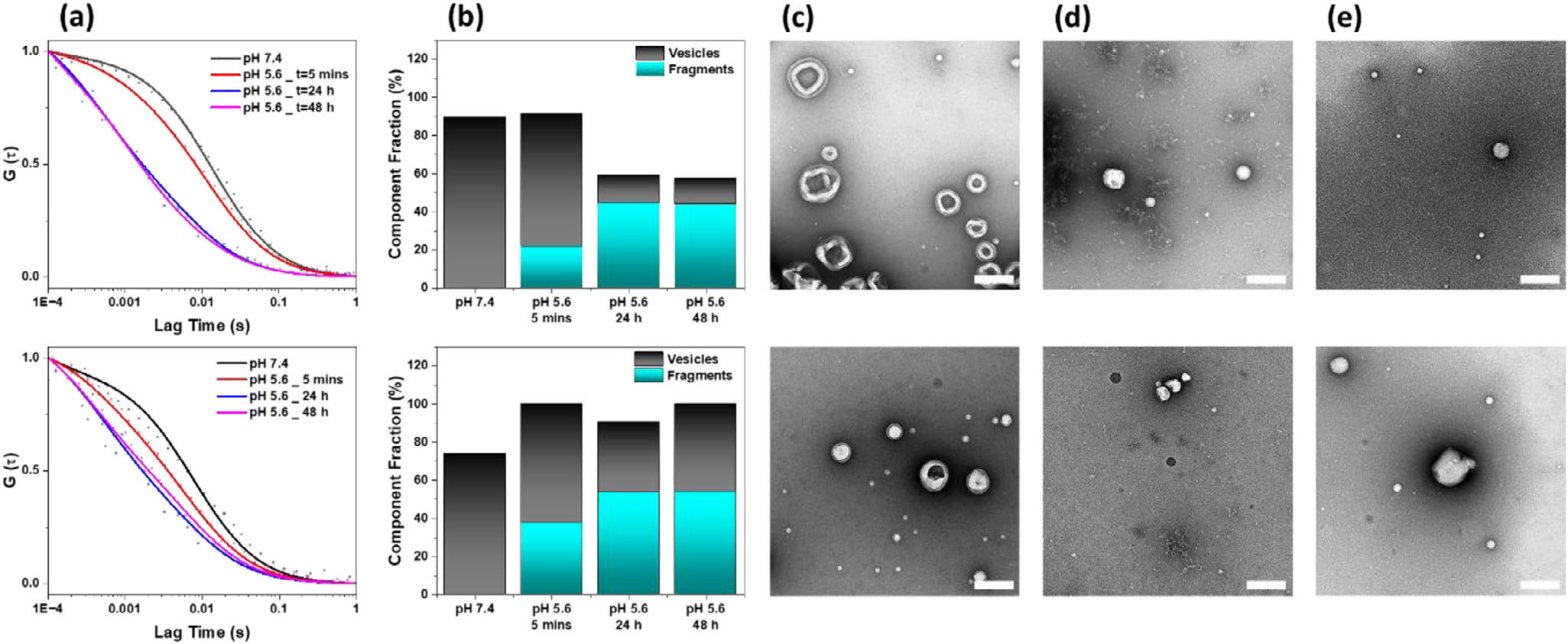
pH‐dependent disintegration of prop‐PMOXA_10_‐*b*‐PDPA_16_ (top) and prop‐PMOXA_21_‐*b*‐PDPA_34_ (bottom) vesicles with Nile Red entrapped in their membrane. (a) Normalized FCS autocorrelation curves of vesicles at pH 7.4 and after exposure to pH 5.6 for different lengths of time. The data represent the mean of 20 independent measurements. (b) Stacked chart shows the percentage of intact vesicles compared to membrane fragments derived from FCS measurements. TEM micrographs were recorded at time points (c) t = 0, pH 7.4, (d) t = 5 min, pH 5.6, and (e) t = 24 h, pH 5.6. All scale bars are 200 nm.

To gain further insight into the release mechanism, the nano‐sized vesicles obtained from prop‐PMOXA_10_‐*b*‐PDPA_16_ and prop‐PMOXA_21_‐*b*‐PDPA_34_ were analyzed by a combination of FCS and TEM to elucidate the effects of changing the pH from 7.4 to 5.6 on their membrane morphology (Figure 7). A hydrophobic dye, Nile Red, was entrapped in the membrane during the self‐assembly process to allow real‐time monitoring of membrane changes. The FCS autocorrelation curves were fitted using a three‐component model to account for the simultaneous presence of intact vesicles, fragments of membranes from disrupted vesicles, and free dye (Equation 4). FCS measurements of PMOXA_10_‐*b*‐PDPA_16_ and prop‐PMOXA_21_‐*b*‐PDPA_34_ vesicles with Nile Red entrapped in their membrane indicated intact vesicles that retained their architecture under neutral conditions (Figure 7a, black curve). The presence of a high percentage of intact vesicles (Figure 7b) was confirmed by ultrastructural TEM analysis (Figure 7c). However, decreasing the pH to 5.6 triggered immediate membrane disruption, as indicated by the marked change in the FCS autocorrelation curves (Figure 7a, red curve). Quantification of these changes indicated the presence of membrane fragments with significantly lower diffusion times (Figure 7b). The presence of membrane fragments was indicated by TEM images of vesicles exposed to pH 5.6 for 5 min (Figure 7d; Figures S16 and S17). This rapid response to acidification reflects the pH‐sensitive behavior of the vesicles, driven by the protonation of the PDPA core, which disrupts hydrophobic interactions and destabilizes the membrane. Over time, the vesicle fragments further disintegrated or aggregated into smaller micellar‐like structures, as revealed by TEM analysis after 24 h (Figure 7e; Figures S16 and S17). Correspondingly, FCS analysis demonstrated a significant increase in the percentage of membrane fragments relative to intact vesicles after 24 h, reflecting the progressive structural changes (Figure 7b). Vesicles of prop‐PMOXA_10_ *‐b‐*PDPA_16_ copolymer exhibited a higher extent of fragmentation compared to prop‐PMOXA_21_ *‐b‐*PDPA_34_ vesicles, which is consistent with their thinner membranes. The increased cargo release efficiency represents a key consideration in further developing these vesicles for biomedical applications. Despite the differences in the rate and extent of disintegration, both vesicle systems apparently reached complete disassembly after 24 h (Figure 7e; Figures S16 and S17 and Table S4). The stepwise pH‐driven disassembly of prop‐PMOXA‐*b*‐PDPA vesicles involves thinner membranes that enable greater disruption and enhanced cargo release efficiency.

**FIGURE 8:**
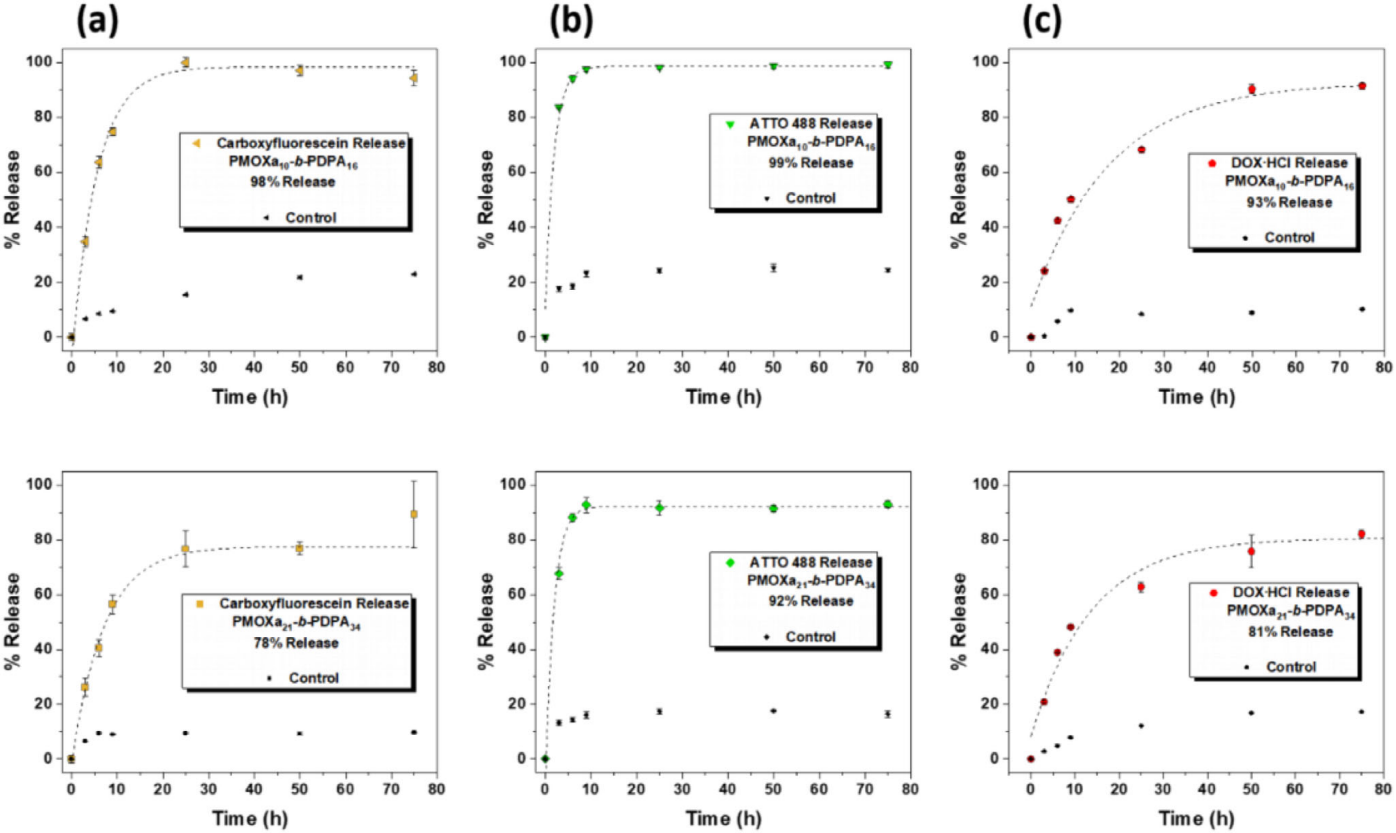
Time‐dependent release of (a) carboxyfluorescein, (b) ATTO 488, and (c) DOX∙HCl from prop‐PMOXA_10_‐*b*‐PDPA_16_ (top) and prop‐PMOXA_21_‐*b*‐PDPA_34_ (bottom) vesicles. All cargo release was conducted at pH 5.6 and the controls were at pH 7.4. The error bars indicate the SD from the fluorimetry measurements.

To quantitatively measure the cargo release kinetics and probe the impact of membrane thickness on release efficiency, we measured the release of carboxyfluorescein, ATTO 488 and DOX∙HCl from prop‐PMOXA_10_‐*b*‐PDPA_16_ and prop‐PMOXA_21_‐*b*‐PDPA_34_ vesicles by using fluorescence spectroscopy (Figure 8). We selected these cargos because their MW falls outside the range that readily diffuses across prop‐PMOXA_10_‐*b*‐PDPA_16_ membranes, ensuring that they are not released uncontrollably (Figure S14).

In contrast to the FCS and TEM studies, where the steep drop in pH resulted in a rapid vesicle disruption, we carried out a kinetics study using cargo‐loaded vesicles that were slowly dialyzed from pH 7.4 to 5.6. We removed aliquots at regular time intervals to evaluate the change in fluorescence intensity of the cargos over time. We hypothesized that slower acidification would not only result in a more gradual swelling of the vesicles, which is expected to slow fragmentation kinetics, but also better reflect the nanocarrier behavior in a biological environment with a less extreme pH gradient. All cumulative release values were calculated according to a one‐component exponential decay function (Equation S1).

Interestingly, the chemical nature of the cargo plays a significant role in the kinetics and efficiency of release from the vesicles. ATTO 488 (MW = 804 Da) showed the fastest release despite having the largest MW, taking approximately 10 h to reach their maximum release for both copolymer vesicles. This is unexpectedly fast compared to carboxyfluorescein (MW = 376 Da), which requires 30–35 h for the maximum release. This extended time for release could possibly be due to the carboxyfluorescein equilibrium between the “open” fluorescent and “closed” non‐fluorescent isomers [31]. When in the open conformation, the carboxyfluorescein can also undergo (de)protonation reactions, which introduce negative charges to the molecule. These charges will induce ionic interactions with the positive PDPA, resulting in a hindered permeation through the membrane and possible adsorption to the resulting membrane fragments, limiting its release efficiency. DOX∙HCl (MW = 560 Da) had by far the slowest release, requiring around 75 h to a maximum release for both polymers. A possible explanation for this significantly slower release from the nanocarriers is the clustering of DOX∙HCl molecules in the aqueous medium [30].

The copolymer composition significantly influenced both the release kinetics and efficiency. The vesicles formed from the larger copolymer showed slower release kinetics and an overall reduced release efficiency. This behavior emphasizes the need for a well‐controlled polymerization where the block lengths and thus, the membrane thickness of the vesicles are minimized. The increased entanglement of the longer copolymer chains restricts the diffusion of protons through the membrane, which slows down the vesicle swelling and membrane fragmentation, resulting in slower release kinetics and a reduced overall delivery efficiency.

#### 2.4.1 Cytotoxicity Assays for Prop‐PMOXA_10_‐b‐PDPA_16_ Vesicles

Finally, we tested whether the prop‐PMOXA_10_‐*b*‐PDPA_16_ vesicles have an adverse effect on the viability of cultured cells by performing cell proliferation assays using HeLa cancer cells (Figure S18). Cells were exposed to different concentrations of prop‐PMOXA_10_‐*b*‐PDPA_16_ vesicles (ranging from 1 mg polymer mL^−1^ to 8 µg polymer mL^−1^) for 24 and 48 h at 37°C. Proliferation of HeLa cells incubated with prop‐PMOXA_10_‐*b*‐PDPA_16_ vesicles was comparable to that of untreated control cells, indicating that the vesicles were not toxic up to 1 mg polymer mL^−1^ at 37°C for at least 48 h, in agreement with usual concentrations used for therapeutic purposes. The absence of intrinsic cytotoxicity supports further development of our vesicles as stimuli‐responsive drug delivery systems in pathologic conditions associated with a slightly acidic environment, as for example tumor microenvironments.

## 3 Conclusions

In this study, we successfully optimized the synthesis of PMOXA‐*b*‐PDPA diblock copolymers and obtained carriers with thinner membranes, ideal for encapsulation and stimuli‐responsive release of cargos. In this respect, we modified the synthesis conditions and used a more versatile in‐house synthesized TTC RAFT agent to prevent macroinitiator hydrolysis at low MWs and enable precise control over the polymerization process producing copolymers with low MWs. In addition, we functionalized the hydrophilic block for the attachment of various molecules upon the formation of supramolecular assemblies to support further targeting or immobilization applications. The copolymers self‐assembled in vesicles with sizes ranging from nanometer up to few microns and having thin membranes. Our findings demonstrate that the PMOXA‐*b*‐PDPA membranes are impermeable to large molecules, while they are permeable to very small molecules (MW ≈ 200 Da). We suggest that other cargos, including the pharmaceutically relevant doxorubicin hydrochloride, are inherently impermeable to the PMOXA‐*b*‐PDPA membranes, making them well‐suited for encapsulation and pH‐triggered release. Additionally, cargo release is not solely driven by membrane permeability changes upon protonation. Instead, our results indicate that vesicle swelling and fragmentation upon pH‐change from neutral to slightly acidic also play a significant role. We observed that lower membrane thickness was correlated with an increased vesicle fragmentation, which in turn enhanced the release kinetics and the cumulative release. These findings highlight the importance of precisely controlling polymer MW to optimize nanocarriers performance, ensuring efficient pH‐triggered cargo release within an appropriate timescale.

## 4 Materials and Methods

### 4.1 Materials

All materials were purchased from Sigma Aldrich or Fischer Scientific and used as received unless otherwise stated. 2‐Methyl‐2‐oxazoline (MOXA, 98%) was distilled prior to use and stored in the glovebox. Phosphate‐buffered saline (PBS) and fetal bovine serum (FBS) were purchased from BioConcept (Switzerland). Dulbecco′s modified Eagle′s medium (DMEM, 4.5 g L^−1^ glucose, with Glutamax‐I) and penicillin/streptomycin 100x stock solution (Gibco) were purchased from Life Technologies Europe. CellTiter 96 AQueous One Solution Cell Proliferation Assay (MTS) was purchased from Promega. 3‐azido‐1‐propanamine was synthesized according to the procedure presented in detail in the Supporting Information, and its chemical structure was confirmed by ^1^H NMR spectroscopy (Figure S1).

### 4.2 Methods

#### 4.2.1 Nuclear Magnetic Resonance Spectroscopy (NMR)

^1^H NMR spectra were recorded at 295 K in a variety of solvents on a Bruker Avance III NMR spectrometer (500 MHz). The instrument was equipped with a direct observe 5 mm BBFO smart probe. Each sample was measured with the default number of 16 scans unless otherwise stated. All spectra were processed with MestReNova software, and chemical shifts were reported in ppm.

#### 4.2.2 Gel Permeation Chromatography (GPC)

SEC measurements were run on an SEC device equipped with three GRAM columns (100 Å, 8 × 300 mm, 10 µm, Agilent Technologies), an Agilent precolumn (GRAM, 8 × 50 mm, 10 µm) and a refractive index (RI) detector (Agilent 1260 Infinity II, USA). Poly(ethylene glycol), poly(methyl methacrylate) and polystyrene MW standards of narrow dispersities were used for calibration. The measurements were performed in HPLC grade DMF (Scharlau, Germany) at 60°C with a flow rate of 1 mL min^−1^. All elugrams were analyzed using WinGPCUniChrom software (version 8.33, PSS).

#### 4.2.3 Differential Scanning Calorimetry (DSC)

DSC curves were recorded on a DSC 214 Polyma (Netzsch, Germany) under inert atmosphere (N_2_). All runs were performed at temperatures ranging from −25°C to 120°C with a heating rate of 10°C min^−1^ and a cooling rate of 40°C min^−1^. All thermograms were analyzed using Proteus 7.1 software (Netzsch).

#### 4.2.4 Dynamic Light Scattering (DLS)

Multiple‐angle light scattering was measured using a LS spectrometer (LS Instruments, Switzerland) equipped with a 633 nm He‐Ne laser (21 mW), AVL correlator and a thermostat (Julabo CF31, Japan). The data was recorded and processed using LS Lab (version 1.8.0.64). Samples were diluted to approximately 0.05 mg mL^−1^ to minimize multiple scattering and then measured at scattering angles between 20° and 150° at 25°C.

#### 4.2.5 Nanoparticle Tracking Analysis (NTA)

NTA measurements were performed using a NS300 (Malvern Panalytical) instrument and analyzed using the NTA software (version 3.4.4, Malvern Panalytical). The self‐assemblies were diluted until approximately 40 particles were detected per frame in the software. The diluted samples were injected with a flow rate of 100 µL min^−1^ at 25°C. The diffusion of the assemblies was recorded over 60 s and measured in triplicate. The hydrodynamic radii of the assemblies were calculated from the average diffusion coefficients using the Stokes–Einstein equation.

#### 4.2.6 Zeta Potential

Zeta potential measurements were performed using a Zetasizer Nano ZSP (Malvern Panalytical) and the data was processed using the Zetasizer software (Malvern Panalytical, version 7.13). The self‐assembles were undiluted after dialyzing into Milli‐Q water and 500 µL were added to a cuvette. The reported zeta potentials are the mean of 3 measurements and the standard deviations are included.

#### 4.2.7 Fluorescence Correlation Spectroscopy (FCS)

An inverted laser scanning confocal microscope (LSM 880, Carl Zeiss, Germany) with a water immersion objective (Zeiss C/Apochromat, *M* = 40, NA = 1.2) was used for FCS. ATTO 488 and DOX∙HCl were excited with an argon laser (λ = 488 nm, MBS 488 filter) and the pinhole size (1 Airy Unit (AU)) was adjusted before recording autocorrelation curves of the free fluorophores.

On a 0.15 mm thick glass coverslip, 20 µL of the free fluorophores or loaded vesicles were placed and the fluorescent fluctuations were measured over time. ZEN software was used to process and analyze raw data. To obtain the fluorescence fluctuation data over time, the samples were recorded for a total of 20 cycles, with each cycle lasting for 10 s. The one‐component diffusion model (Equation 1) was used to fit the experimental autocorrelation curves for free ATTO 488 and DOX∙HCl in PBS.

(1) $$G\left(\tau \right)=1+\left(1+\frac{T}{1-T}e^{-\left(\frac{\tau }{\tau_{trip}}\right)}\right)\frac{1}{N}\left[\frac{1}{1+\frac{\tau }{\tau_{D}}\sqrt{1+R^{2}\frac{\tau }{\tau_{D}}}}\right]$$

where G(*τ*) is the autocorrelation function, *N* is the average number of particles in the observation volume, *τ*_D_ the diffusional correlation time and *R* the structural parameter (5). *T* is the fraction of molecules in triple state and *τ*_trip_ is the triplet time. The diffusion coefficient *D* was calculated as in Equation (2), using the relation between the x–y dimension of the confocal volume (*ω*_xy_) and *τ*_D_:

(2) $$\tau_{D}=\frac{\omega_{xy^{2}}}{4D}$$

The two‐component diffusion model (Equation 3) was used for fitting the experimental autocorrelation curves for the encapsulated ATTO 488, DOX∙HCl and Nile red used to stain the membrane of the nanoassemblies; as well as to check the fraction of free components that may be present in solution after purification and were not encapsulated.

(3) $$\begin{array}{ccc} G\left(\tau \right) & = & 1+\left(1+\frac{T}{1-T}e^{-\left(\frac{\tau }{\tau_{trip}}\right)}\right)\frac{1}{N}\left[\frac{f_{1}}{1+\frac{\tau }{\tau_{D1}}\sqrt{1+R^{2}\frac{\tau }{\tau_{D1}}}}\right]\\ & & \;+\frac{1}{N}\left[\frac{f_{2}}{1+\frac{\tau }{\tau_{D2}}\sqrt{1+R^{2}\frac{\tau }{\tau_{D2}}}}\right] \end{array}$$

where T is the fraction of molecules in the triplet state; *τ*_trip_ is the triplet state relaxation time, *N* is the average number of particles in the observation volume, $f_{1}$ and $f_{2}$ are the particle fractions ($f_{1}+f_{2}=1)$; $\tau_{D_{1}}$, $\tau_{D_{2}}$ are the diffusion times of the corresponding component 1 or 2 and *R* is the structural parameter (5).

The three‐component diffusion model (Equation 4), including the triplet state, was used to fit the experimental autocorrelation curves to monitor the release of molecules, specifically the encapsulated ATTO 488, DOX∙HCl, and Nile red (used to stain the membrane) from the nanoassemblies. This model was applied both before and after acidification to monitor the release of the components over time.

(4) $$\begin{array}{cc} G\left(\tau \right) & =1+\left(1+\frac{T}{1-T}e^{-\left(\frac{\tau }{\tau_{trip}}\right)}\right)\frac{1}{N}[\frac{f_{1}}{1+\frac{\tau }{\tau_{D1}}\sqrt{1+R^{2}\frac{\tau }{\tau_{D1}}}}\\ & \;+\frac{f_{2}}{1+\frac{\tau }{\tau_{D2}}\sqrt{1+R^{2}\frac{\tau }{\tau_{D2}}}}+\frac{f_{3}}{1+\frac{\tau }{\tau_{D3}}\sqrt{1+R^{2}\frac{\tau }{\tau_{D3}}}}] \end{array}$$

where $f_{1},f_{2}$ and $f_{3}$ are the particle fractions ($f_{1}+f_{2}+f_{3}=1).$

The Einstein–Stokes Equation (5) was used to calculate the hydrodynamic radius (*R*_h_) of the loaded vesicles, where *D* is the diffusion coefficient, *k*_B_ the Boltzmann's constant, *T* the absolute temperature and *η* the viscosity of the medium:

(5) $$D=\frac{k_{B}T}{6\pi \eta R_{h}}$$

#### 4.2.8 Fluorimetry

Fluorimeter measurements were performed using a spectrofluorometer (Jasko, FP‐8200) equipped with a Xe lamp and the emission intensity was measured by fixed wavelength detection. The data was recorded using Spectra Manager (version 2.14.02). Each measurement cycle had a 5 s interval and was repeated for five cycles with high sensitivity. The ATTO 488 loaded samples were measured using an excitation wavelength of 499 ± 1.25 nm and an emission wavelength of 520 ± 1.25 nm. The DOX∙HCl loaded samples were measured using an excitation wavelength of 480 ± 5 nm and an emission wavelength of 590 ± 10 nm. The carboxyfluorescein‐loaded samples were measured using an excitation wavelength of 493 ± 2.5 nm and an emission wavelength of 517 ± 2.5 nm.

#### 4.2.9 Transmission Electron Microscopy (TEM)

For TEM analysis, 5 µL of the sample solution was adsorbed to a discharged 400‐mesh copper grid, blotted, and negatively stained with 2% Phosphotungstatic acid (PTA) at pH 7. Images were recorded on a Thermo Fisher Scientific Talos L120C TEM at 80 kV acceleration voltage.

#### 4.2.10 Cryo‐Transmission Electron Microscopy (Cryo‐TEM)

Cryo‐TEM images were recorded on a Talos electron microscope (Thermo Fisher, USA) equipped with a CETA camera using a Gatan 626 Cryo‐holder. 4 µL of a 1 mg mL^−1^ self‐assembly dispersion was adsorbed onto a holey carbon‐coated grid (Lacey, Tedpella, USA). The sample was vitrified into liquid ethane using a Leica GP plunger (Leica, Austria). An acceleration voltage of 200 kV and a nominal magnification of 57 000× was used. Membrane thicknesses of the imaged vesicles were measured using ImageJ (NIH, USA, version 1.54f).

#### 4.2.11 Cell Culture

HeLa cells (cervical adenocarcinoma, human) were routinely subcultured in DMEM supplemented with penicillin/streptomycin and 10 % FBS (DFBS). Cells were maintained at 37°C in a humidified atmosphere containing 5% CO_2_ for up to 20 passages.

#### 4.2.12 Cell Proliferation Assay

The effect of polymersomes on the viability of cultured cells was examined using the colorimetric CellTiter 96® AQ_ueous_ One Solution Cell Proliferation Assay (Promega). In brief, 3 × 10^−3^ HeLa cells were plated in 100 µL DFBS/well in a clear‐bottom 96‐well plate and cultured for 24 h. Then, the medium was replaced by different concentrations of polymersomes in PBS to DFBM at a volume ratio of 1:3. To establish a negative control, cells were incubated with PBS/DFBS lacking polymersomes, and background fluorescence was determined from samples containing PBS/DFBM without cells. After 24 and 48 h incubation, cells were rinsed twice with PBS, and 100 µL fresh DFBS were added to each well. 20 µL CellTiter 96® AQueous One Solution Reagent were added directly to culture wells and incubated for 1–2 h before the absorbance at 490 nm was recorded with a 96‐well plate reader (SpectraMax iD5 microplate reader; Molecular Devices, US). Obtained values, after background subtraction, were normalized to wells with untreated cells. All proliferation experiments were carried out in quadruplicate and repeated three times. Histograms present average cell viability with error bars representing standard deviation.

### 4.3 Synthesis

#### 4.3.1 Prop‐PMOXA_n_‐OH Synthesis

Prop‐PMOXA_n_‐OH was synthesized according to a modified procedure [32]. Briefly, a microwave vial containing propargyl *p*‐toluenesulfonate, 2‐methyl‐2‐oxazoline and acetonitrile was prepared and sealed in the glovebox. The monomer concentration was set to 3 M and the monomer‐to‐initiator molar ratio was varied according to the target block length (for *n* = 6, 10, 16, and 21, a monomer‐to‐initiator molar ratio of 5, 10, 15, and 20, respectively, was used). The solution was heated to 100°C for 30 min to reach full monomer conversion. The reaction was then quenched with excess Milli‐Q water, the vial was opened and the crude prop‐PMOXA_n_‐OH was purified by precipitation in Et_2_O.

50 µL aliquots were taken under a nitrogen flow at regular intervals and mixed into D_2_O for ^1^H NMR kinetics. The aliquot measurements were all normalized according to the tosylate aromatic peaks and the reduction of the monomer peaks at 3.79 and 4.21 ppm were monitored over time.

#### 4.3.2 4‐Cyano‐4‐(((ethylthio)carbonothioyl)thio)pentanoic Acid RAFT Agent (TTC) Synthesis

TTC was synthesized using a modified procedure, as reported by Khalid Ferji et al. [33]. Sodium ethanthiolate (4.2 g, 45 mmol, 1 eq) was dissolved in Et_2_O (100 mL, anhydrous) and cooled to 0°C. Carbon disulfide (3.4 g, 45 mmol, 1 eq) was added dropwise then the mixture was stirred for 2 h at 0°C. Iodine (5.9 g, 23 mmol, 1.02 eq) was added portion‐wise and allowed to mix for a further 2 h at room temperature. Sodium iodide was removed by vacuum filtration and the volatiles were evaporated, producing a concentrated red oil, bis(ethylsulfanylthiocarbonyl) disulfide (Figure S2). The oil was dissolved in EtOAc (80 mL), ACVA (6.19 g, 22.1 mmol, 1.5 eq) was added and the solution was refluxed at 80°C for 16 h. The crude product was washed with Milli‐Q water (x5, 100 mL) and then purified by flash chromatography to yield the pure product (9.608 g, 81% yield). The chemical structure of the TTC RAFT agent was confirmed by ^1^H NMR spectroscopy (Figure S3).

#### 4.3.3 Prop‐PMOXA_n_‐CTA Macroinitiator Synthesis

Prop‐PMOXA_n_‐CTA macroinitiators were synthesized via Steglich esterification. DCC (3 eq) was dissolved in DCM and cooled to −20°C. Prop‐PMOXA_n_‐OH (1 eq), TTC (3 eq), and DMAP (0.25 eq) were dissolved in DCM and cooled to 0°C. The DCC solution was then added dropwise and the mixture was allowed to stir for 2 days while warming to room temperature.

#### 4.3.4 Prop‐PMOXA_n_‐b‐PDPA_m_ Photo‐RAFT Polymerization

Prop‐PMOXA_n_‐CTA (1 eq), 2‐(diisopropylamino)‐ethyl‐methacrylate (m eq) was dissolved in CHCl_3_ in a 20 mL transparent vial with a monomer concentration of 1 M and a total volume of 17 mL. For m = 11, 16, 30, and 34, a monomer‐to‐initiator molar ratio of 10, 15, 25, and 30 was used, respectively. The glass vial was sealed with a rubber stopper, bound with parafilm and the solution was degassed via nitrogen bubbling for 5 min, then heated to 40°C and irradiated with a near‐UV LED lamp (405 nm) until full monomer conversion was achieved. All volatiles were evaporated from the solution. The crude polymer was then dissolved in EtOH and purified by dialysis (RC membrane, MWCO = 1 kDa). The pure product was dried on the Schlenk line and characterized using ^1^H NMR spectroscopy.

Regular aliquots were taken under a nitrogen stream and dissolved in CDCl_3_ for ^1^H NMR measurements. The spectra were normalized against the PMOXA backbone and the methacrylate peaks reduction was monitored over time.

#### 4.3.5 Diblock Click Reactions

Prop‐PMOXA_16_ *‐b‐*PDPA_42_ (50 mg, 4.72 µmol, 1 eq) was dissolved in DMF (500 µL, anhydrous), then Cu(I)Br (0.34 mg, 2.36 µmol, 0.5 eq), PMDETA (0.50 µL, 2.36 µmol, 0.5 eq) and the azide (47.2 µmol, 10 eq) were added sequentially. The reaction vessel was sealed with a rubber septum and purged with nitrogen for 10 min, which was then stirred for 4 days at room temperature. The crude product was purified by dialysis (RC, MWCO 1 kDa, EtOH) and the product was characterized by ^1^H NMR.

### 4.4 Supramolecular Self‐Assembly

#### 4.4.1 Solvent Switch Self‐Assembly

All diblocks were self‐assembled using the solvent switch method such that the final concentration of the self‐assemblies was always 5 mg mL^−1^. Namely, 10 mg of polymer was weighed into a 10 mL round‐bottomed flask and then dissolved in 400 µL of EtOH. 1.6 mL of PBS was then added at a rate of 10 µL min^−1^ under stirring at 300 rpm. The suspension was then purified by dialysis (regenerated cellulose, MWCO 1 kDa, PBS) against PBS to remove residual small molecules, centrifuged (5000 rpm, 10 min) and size exclusion chromatography.

#### 4.4.2 Size Exclusion Chromatography (SEC)

SEC purification of self‐assemblies was performed using an ÄKTA Go protein purification system (Cytiva). The column was packed with a dextran‐agarose composite matrix (bead dimensions = 10 × 300 mm) equilibrated in PBS. Dialyzed samples were fractionated and visualized using the UNICORN software (version 7.8), where fractions containing aggregates and smaller impurities were removed. Fractions containing self‐assemblies were pooled to give the final self‐assembly suspension.

#### 4.4.3 Giant Unilamellar Vesicle (GUV) Self‐Assembly by Microfluidics

GUVs were formed from double‐emulsion templates generated using a microfluidic chip. For this, an inner aqueous phase, a polymer organic phase carrying the BCPs, and an outer aqueous phase were cross‐flowed on a microfluidic six‐way junction chip. A three‐module low‐pressure syringe pump system (Cetoni neMESYS) was used for flow control of fluid phases in the microfluidic device. Microfluidic chip coating and operation was done according to previously published protocols.3 PBS supplemented with 200 mm sucrose and 5 % w/v PEG (Mn 35’000) was used as an inner aqueous (IA) phase. A 3:2 v/v mixture of hexane and chloroform was used to dissolve the polymers to a final concentration of 4 mg mL^−1^, forming the polymer organic (PO) phase. An outer aqueous (OA) phase consisting of PBS supplemented with 5 % w/v PEG (Mn 35’000), 100 mm NaCl, and 0.1 % v/v Pluronics F‐68 was used for cross‐flowing and breaking off the formed double emulsions. Flow rates of 3, 1, and 50 µL min^−1^ were used to flow the IA, PO, and OA through the microfluidic chips. The produced double emulsions were collected for 10 min in an Eppendorf tube containing 300 µL OA. For the removal of the organic phase and subsequent formation of GUVs, the double emulsion suspension was diluted in OA and exposed to air, thereby evaporating the volatile organic solvents, leaving behind micron‐sized unilamellar vesicles [3, 7].

#### 4.4.4 GUV Permeability

For the membrane permeability to dye, empty GUVs were incubated for 1 h with 5 µm of fluorescent dye in salt form (resorufin, carboxyfluorescein, sulforhodamine B, calcein, or ATTO 488), then imaged using confocal laser scanning microscopy. The fluorescent intensity inside the GUVs was obtained by subtracting the background to give the ratio of dye molecules that had permeated through the membrane.

#### 4.4.5 Confocal Laser Scanning Microscopy (CLSM)

GUVs were imaged on a confocal microscope (Zeiss LSM880) equipped with a 20x/0.8 M27 (Plan‐Apochromat) objective. BODIPY 630/650 was used for membrane staining at a concentration of 2.5 µm, and GUVs were typically imaged at 1:10 dilution in OA in an Ibidi µ‐slide. Images were typically recorded with an image size of 1024 × 1024 pixels with the pinhole set to 1 Airy unit and the bit depth set to 16 bit. Images were analyzed using Fiji image analysis software and python scripts to measure GUV fluorescence or size.

#### 4.4.6 GUV Stability Determination

For the determination of the stability of GUVs, the IA was supplemented with 10 µm ATTO 488 and the 11 µL of the GUV suspension were added to a urinalysis slide (Fast Read 102 counting chambers) and imaged on an Olympus EP50 microscope. GUVs were counted from the images and normalized to the number of GUVs after production (day 0).

#### 4.4.7 Statistical Analysis

A one‐way analysis of variance (ANOVA) was used to compare fluorescence intensity. *p* < 0.05 was considered statistically significant. The significance level of the calculated *p* values was indicated using asterisks: *p* > 0.05 (n.s.), *p* < 0.05 (*). Statistics were done using the Python scipy.stats library [34].

## Conflicts of Interest

The authors declare no conflict of interest.

## Supporting information




**Supporting File 1**: marc70007‐sup‐0001‐SuppMat.docx.

## Data Availability

The data that support the findings of this study are available in the supplementary material of this article.
