# Supplementary material for: Engineering pH‐Responsive Nanocarriers via an Optimized Synthesis of PMOXA‐b‐PDPA Amphiphilic Diblock Copolymers
Source: Macromol Rapid Commun. 2025 Jul 28;46(20):e00418. doi: 10.1002/marc.202500418 (PMC12536392; doi:10.1002/marc.202500418)
Supplement: Supplementary file 1 — Supporting File 1: marc70007‐sup‐0001‐SuppMat.docx. [file MARC-46-e00418-s001.docx]

Supporting Information

Engineering pH-Responsive Nanocarriers *via* an Optimized Synthesis of PMOXA-*b*-PDPA Amphiphilic Diblock Copolymers

John Peter Coats, Anamarija Nikoletić, Lukas Heuberger, Voichita Mihali, Cora-Ann Schoenenberger, Ionel Adrian Dinu, Cornelia G. Palivan*

J. P. Coats, A. Nikoletić, L. Heuberger, V. Mihali, C.-A. Schoenenberger, I. A. Dinu, C. G. Palivan

Department of Chemistry, University of Basel, BPR1096, Mattenstrasse 22, Basel, 4002 Switzerland
E-mail: [cornelia.palivan@unibas.ch](mailto:cornelia.palivan@unibas.ch)

A. Nikoletić, C. G. Palivan

Swiss Nanoscience Institute, University of Basel, Klingelbergstrasse 82, Basel, 4056 Switzerland

**1. Synthesis of 3-azido-1-propanamine**

**1a. Synthesis method**

3-Bromopropylamine hydrobromide (10 g, 45.7 mmol, 1 eq) and sodium azide (8.906 g, 137 mmol, 3 eq) were dissolved in 250 mL of Milli-Q water and refluxed at 110°C for 16h. All water was evaporated using reduced pressure then redissolved in minimal Milli-Q until a yellow oil appears. Potassium hydroxide (10 g, 178 mmol, 3.9 eq) was dissolved portion wise while cooling in an ice bath. Solid particles were removed by vacuum filtration and the crude product was extracted into Et_2_O (x3, 100 mL). The organic phases were combined and dried over magnesium sulfate. The solid residue was filtered off, and all solvent was removed under reduced pressure producing pure 3-azido-1-propanamine (4.16 g, 41.6 mmol, 91% yield).

**1b. ^1^H NMR characterization**


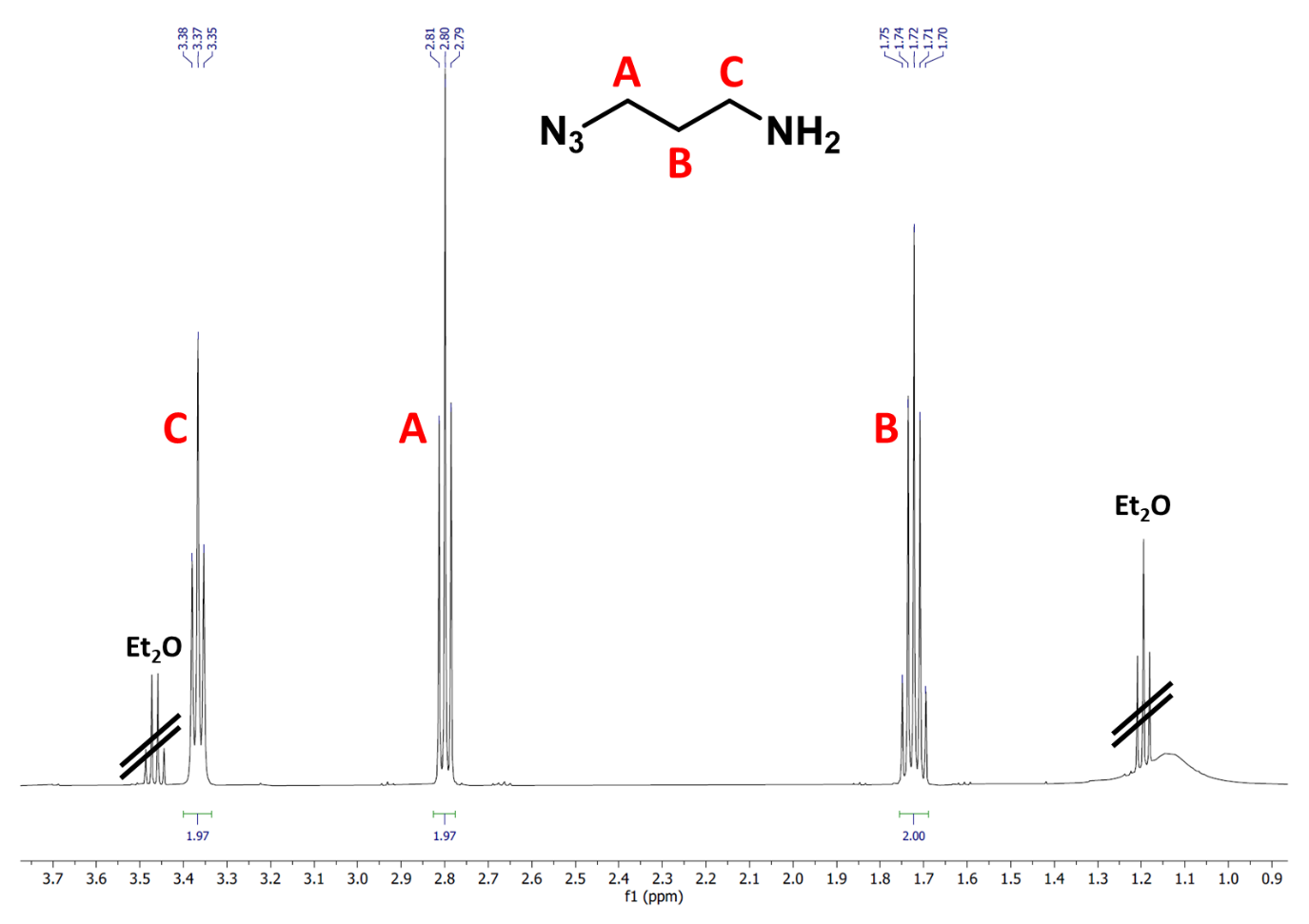


**Figure S1**. ^1^H NMR of 3-azido-1-propanamine in CDCl_3_.

**2. Trithiocarbonate CTA ^1^H NMR Characterization**

**2a.** **Bis(ethylsulfanylthiocarbonyl) disulfide**


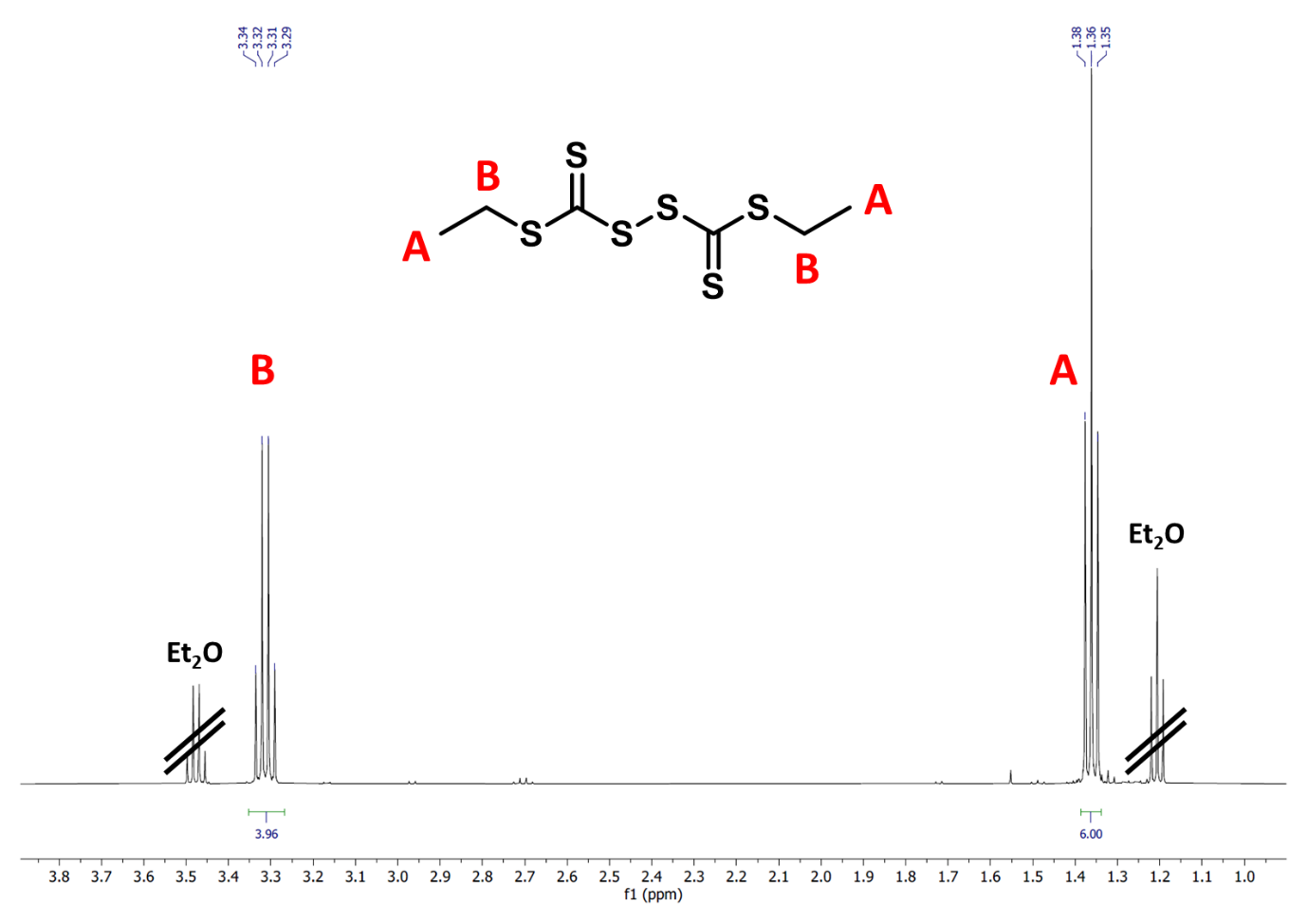


**Figure S2**. ^1^H NMR of bis(ethylsulfanylthiocarbonyl) disulfide in CDCl_3_.

**2b.** **4-Cyano-4-(((ethylthio)carbonothioyl)thio)pentanoic acid**


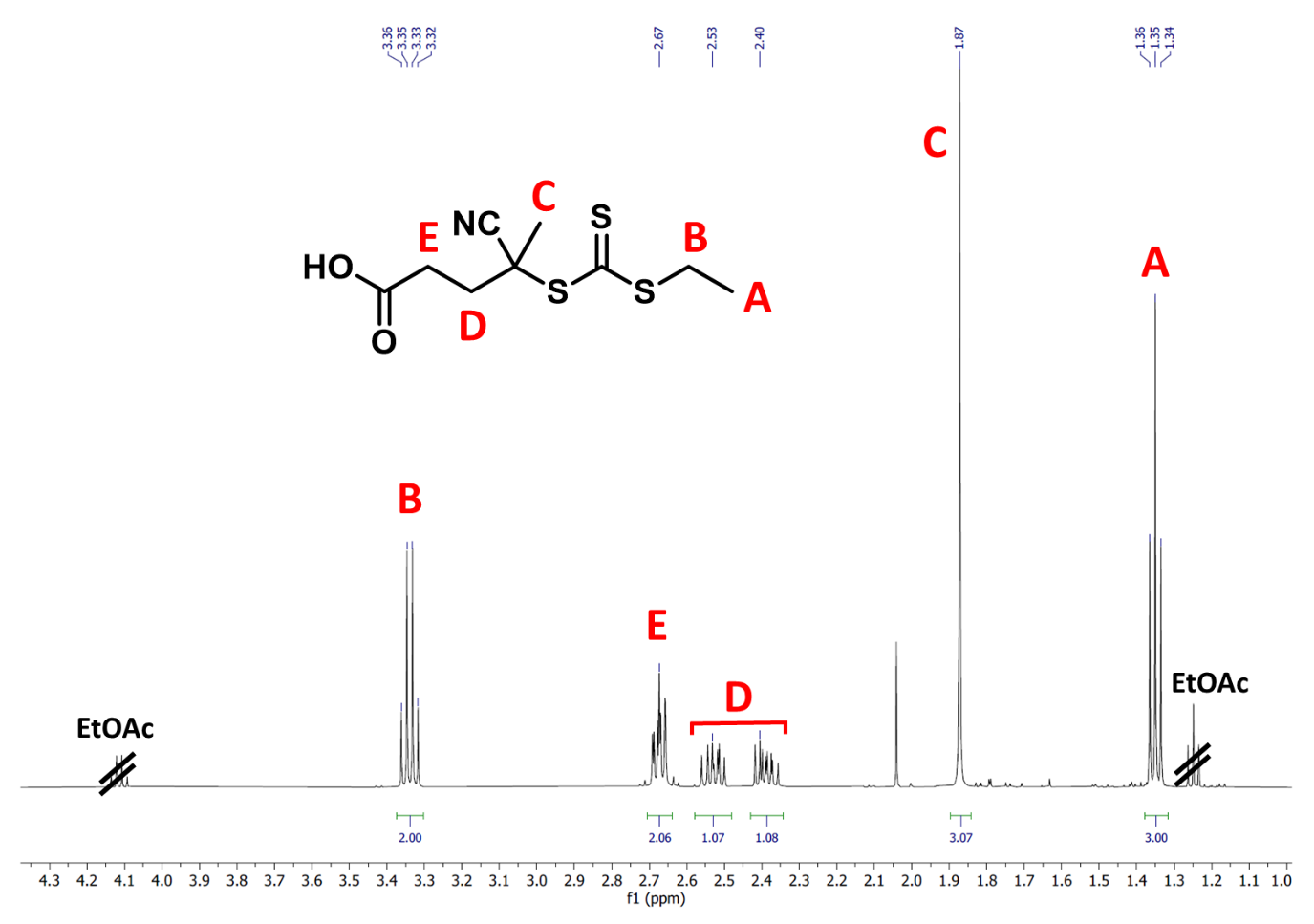


**Figure S3**. ^1^H NMR of 4-cyano-4-(((ethylthio)carbonothioyl)thio)pentanoic acid in CDCl_3_.

**2c. Diblock PI-RAFT Reactions**

The diblock polymerization from the PMOXA-CTA was performed using a homemade photoreactor where the water bath temperature could be precisely controlled with a thermostat and the inner walls of the chamber were mirrored to focus the near-UV light onto the sample, which both minimizes light leakage onto the operator and maximizes the delivered light dosage to the sample. The lamp used was an EvoluChem LED which delivers an irradiance of 201 mW cm^-2^ at a wavelength of 405 nm. To achieve convenient reaction kinetics, the water bath was set to 40 ºC and the sample was subjected to constant irradiation until full monomer conversion was reached. These reaction conditions allowed the preparation of the reaction solutions, full monomer conversion and the dialysis purification setup all within a single day. Aliquots of the reaction mixture were regularly sampled and analyzed using ^1^H NMR spectroscopy to determine the monomer conversion over time. After an initial induction period, the polymerization progressed steadily, achieving full monomer conversion within 5 h.

Near quantitative conversion of the terminal alkyne was achieve via CuAAC, setting a benchmark for tailorable end-group functionality by this method. Alternatively, no further CTA end-group analysis was performed on these polymers since the R- group originating from the CTA sits inside the hydrophobic domain of the membranes after self-assembly. This positioning prevents any useful coupling reactions from advancing our intended cargo encapsulation/release applications.

**3. (Co)polymer characterization**

**3a. ^1^H NMR spectra**


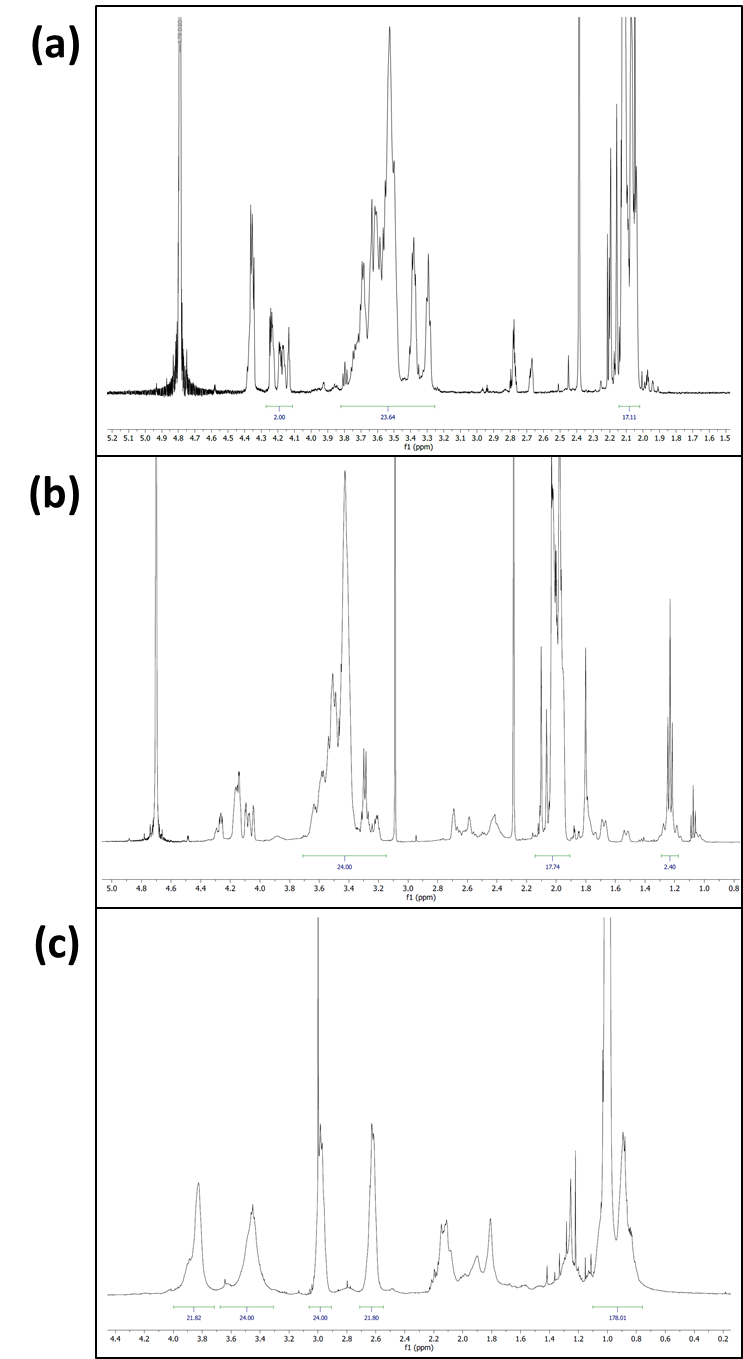


**Figure S4**. ^1^H NMR spectrum of (a) prop-PMOXA_6_-OH in D_2_O, (b) prop-PMOXA_6_-CTA in D_2_O and (c) prop-PMOXA_6_-b-PDPA_11_ in CDCl_3_.


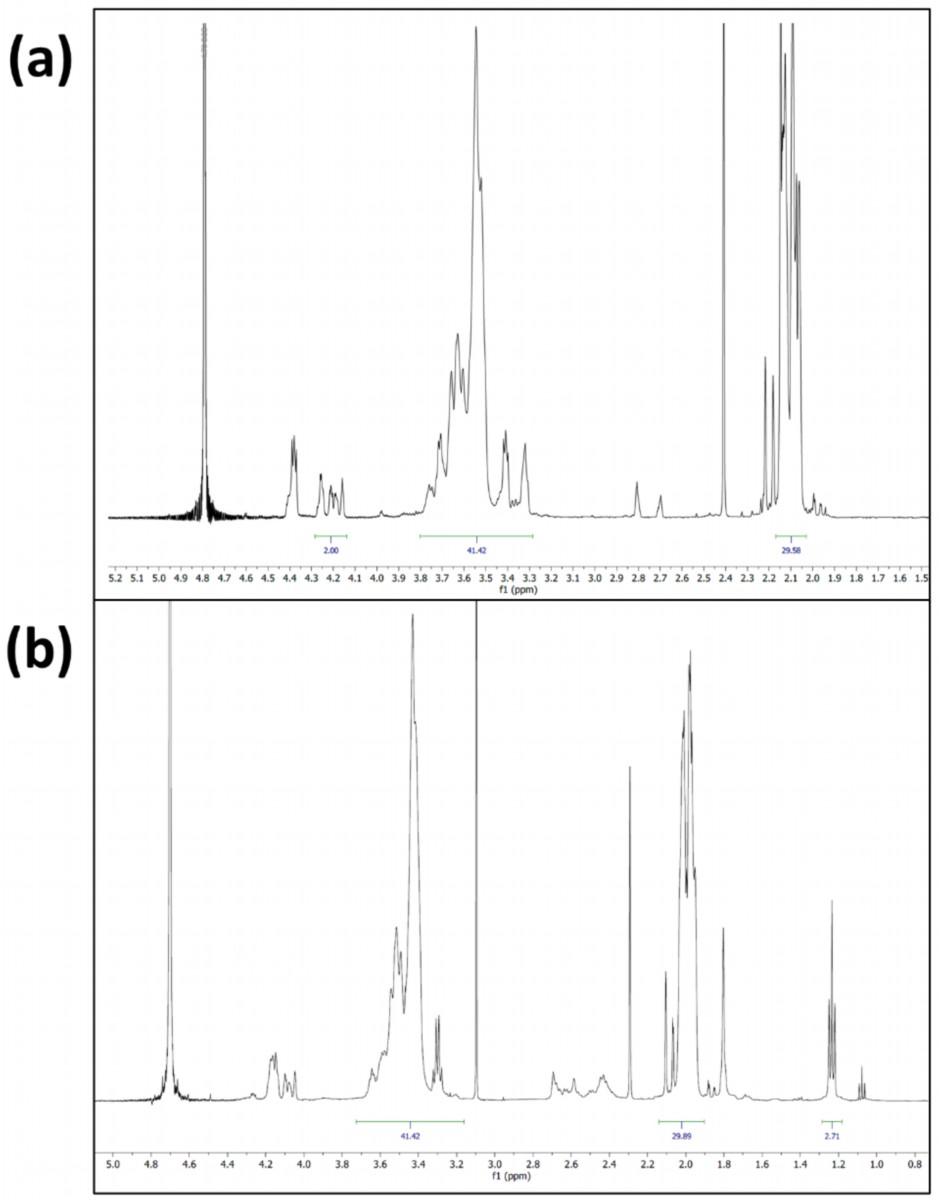


**Figure S5**. ^1^H NMR spectrum of (a) prop-PMOXA_10_-OH in D_2_O and (b) prop-PMOXA_10_-CTA in D_2_O.


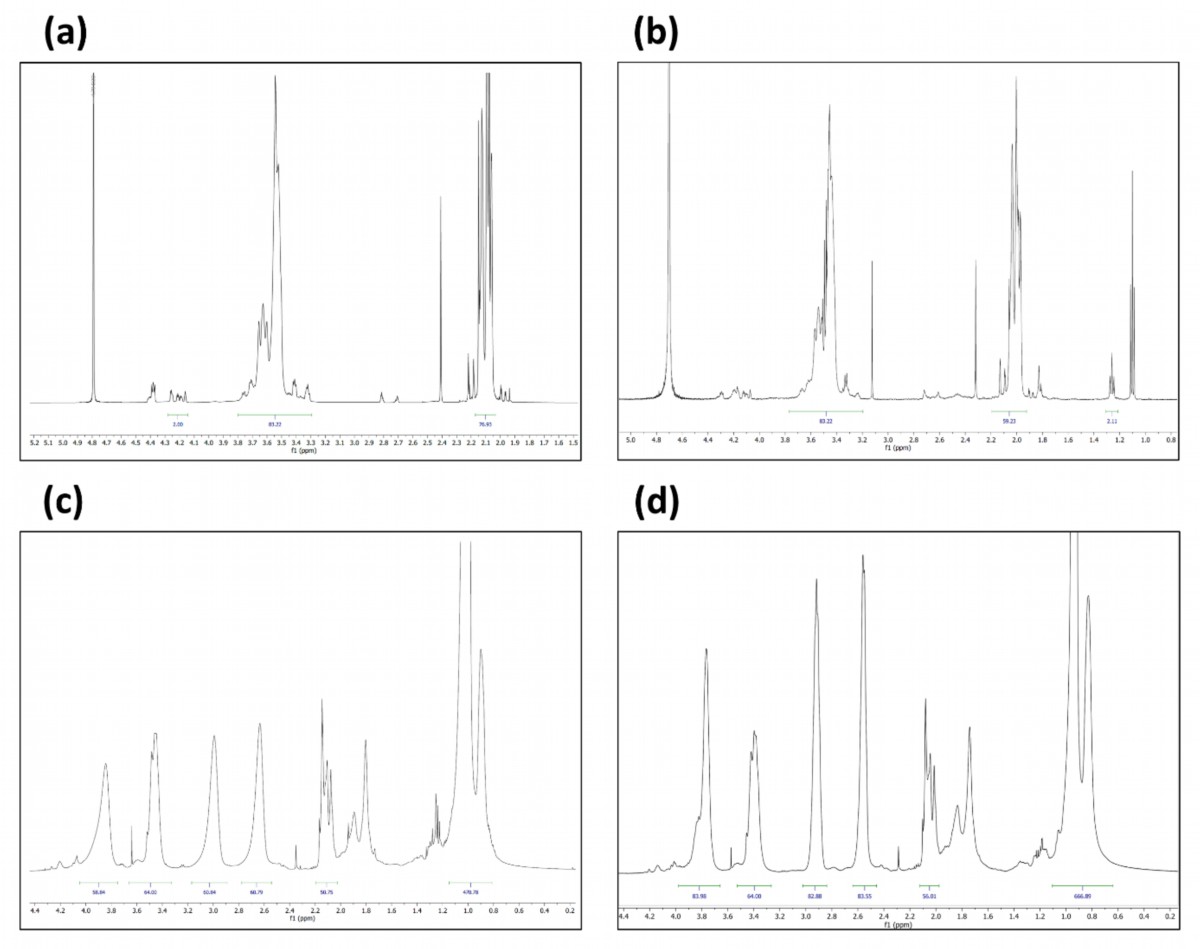


**Figure S6.** ^1^H NMR spectrum of (a) prop-PMOXA_16_-OH in D_2_O, (b) prop-PMOXA_16_-CTA in D_2_O, (c) prop-PMOXA_16_-*b*-PDPA_30_ in CDCl_3_ and (d) prop-PMOXA_16_-*b*-PDPA_42_ in CDCl_3_*.*


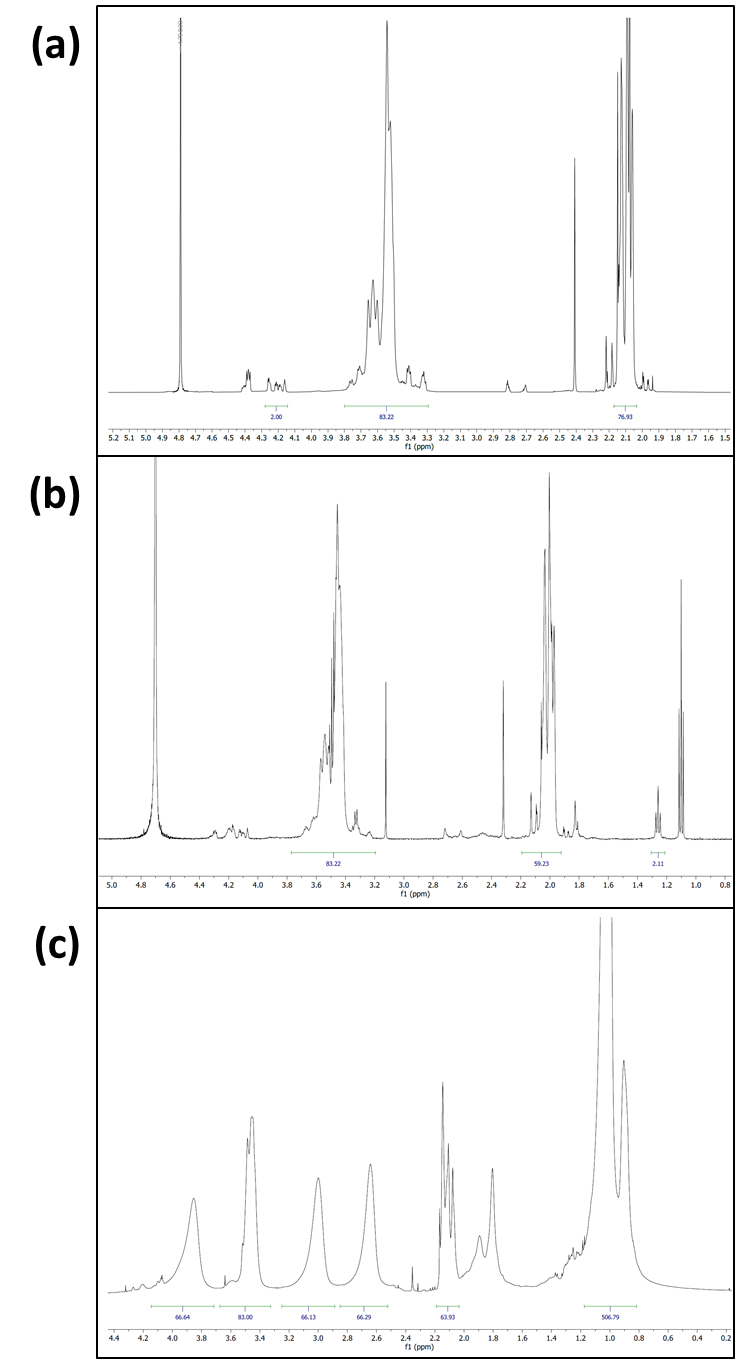


**Figure S7.** ^1^H NMR spectrum of (a) prop-PMOXA_21_-OH in D_2_O, (b) prop-PMOXA_21_-CTA in D_2_O and (c) prop-PMOXA_21_-*b*-PDPA_34_ in CDCl_3_

**3b. GPC elugrams**


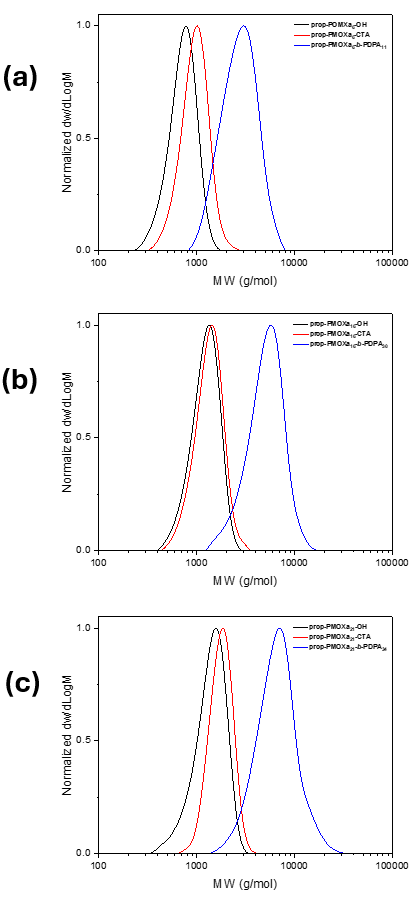


**Figure S8**. GPC analysis showing MW evolution of prop-PMOXA_n_-OH homopolymer, prop-PMOXA_n_-CTA macro-initiator and the resulting prop-PMOXA_n_-b-PDPA_m_ diblock copolymer. (a) prop-PMOXA_6_-b-PDPA_11_, (b) prop-PMOXA_16_-b-PDPA_30_ and (c) prop-PMOXA_21_-b-PDPA_34_.

**3c. DSC thermograms**

**Figure S9**. DSC analysis of diblock copolymers prop-PMOXA_6_-b-PDPA_11_, prop-PMOXA_10_-b-PDPA_16_, prop-PMOXA_16_-b-PDPA_30_ and prop-PMOXA_21_-b-PDPA_34_.

**4. Supramolecular assembly analysis**

**4a. Diblock Self-Assembly and Purification**

All self-assemblies were performed with the solvent switch method where the diblocks were dissolved in ethanol and PBS (pH 7.4) was slowly added dropwise to induce a controlled self-assembly. Some of the crude self-assemblies would show a minor population of larger aggregates in the range of 1-5 µm in the DLS analysis. These were removed by SEC where the desired vesicles were eluted after approximately 6 mL and were detected by a combination of UV absorbance and conductivity.

**4b. DLS size profiles**


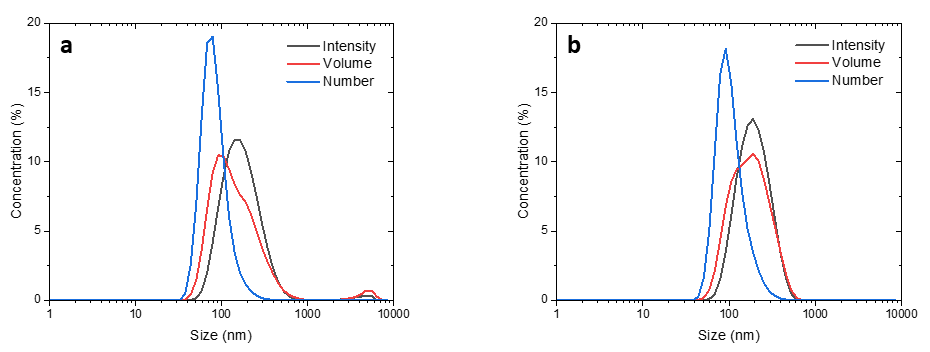


**Figure S10**. DLS analysis of the supramolecular assemblies formed from a) prop-PMOXA_6_-b-PDPA_11_ and b) prop-PMOXA_16_-b-PDPA_30_ after solvent switch self-assembly.

**4c. NTA size profiles**


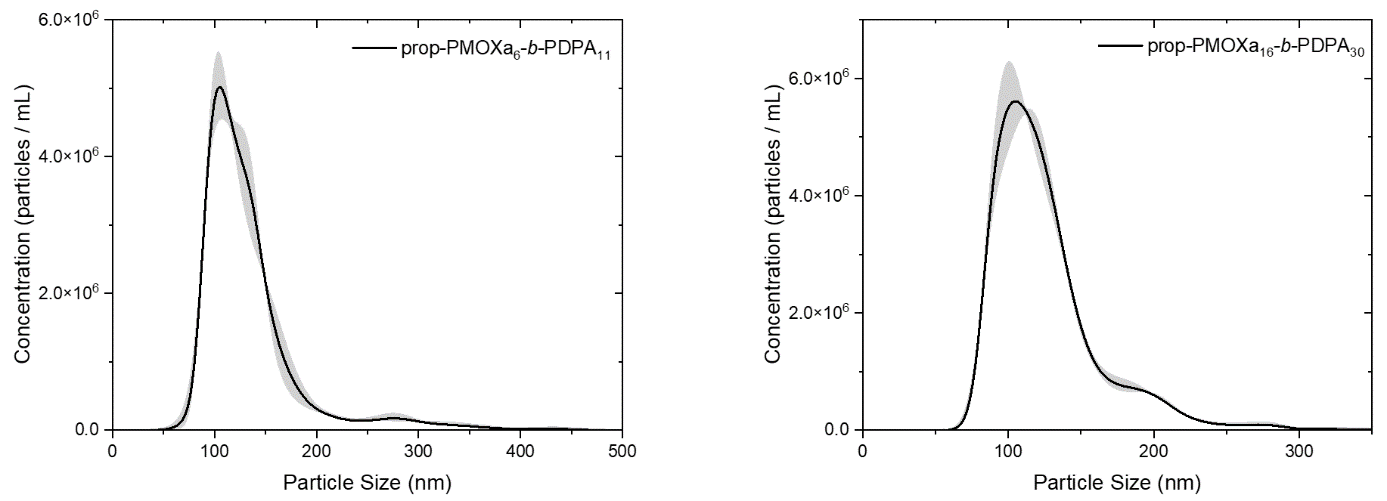


**Figure S11**. NTA analysis of the supramolecular assemblies formed from prop-PMOXA_6_-b-PDPA_11_ and prop-PMOXA_16_-b-PDPA_30_ after solvent switch self-assembly.

**4d. cryo-TEM Micrographs**

**Figure S12.** Cryo-TEM micrographs of vesicles self-assembled from a) prop-PMOXA_6_-*b*-PDPA_11_ and b) prop-PMOXA_10_-*b*-PDPA_16_.


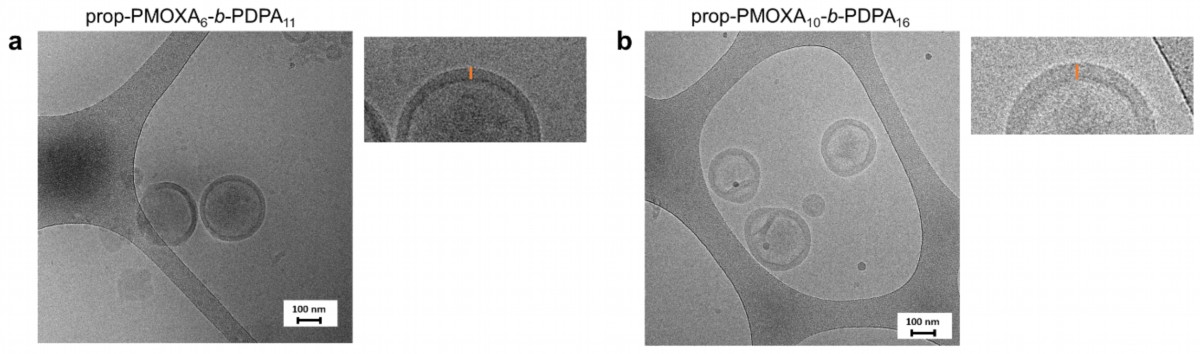


**Figure S13.** Cryo-TEM micrographs of vesicles self-assembled from a) prop-PMOXA_15_-*b*-PDPA_30_ and b) prop-PMOXA_21_-*b*-PDPA_34_


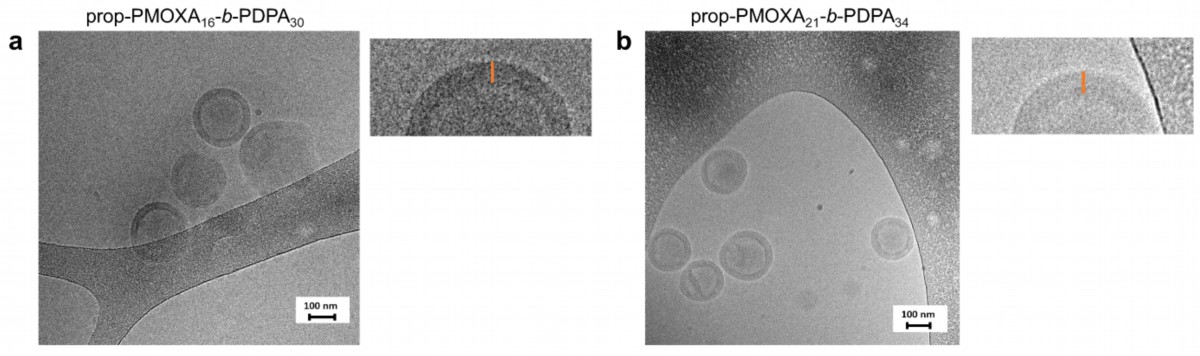


**Table S1.** Characterization data of the self-assemblies obtained from prop-PMOXA_n_-*b*-PDPA_m_ diblock copolymers.

| **Composition** | ***D_h_* (nm)*^a^*** | **PDI** | **⌀ (nm)*^b^*** | ***l*_Membrane_ (nm)*^c^*** |
| --- | --- | --- | --- | --- |
| **PMOXA_6_-*b*-PDPA_11_** | 256 ± 6 | 0.411 | 122 ± 34 | 10.5 ± 1.4 |
| **PMOXA_10_-*b*-PDPA_16_** | 199 ± 4 | 0.251 | 163 ± 65 | 13.8 ± 2.8 |
| **PMOXA_16_-*b*-PDPA_30_** | 172 ± 0.3 | 0.309 | 133 ± 50 | 14.2 ± 2.0 |
| **PMOXA_21_-*b*-PDPA_34_** | 157 ± 10 | 0.149 | 126 ± 38 | 16.7 ± 1.8 |

*^a^*Hydrodynamic diameter (D_h_) determinated from DLS, *^b^*Obtained from NTA, *^c^*Measured from cryo-TEM micrographs.

Our DLS and cryo-TEM data is consistent with established physical models for polymer vesicles. Discher et al. demonstrated that the bending rigidity (κ) of diblock copolymer membranes scales quadratically with membrane thickness, κ ∝ d² (﻿Langmuir 2004, 20, 540-543). As a result, vesicles with thinner, more flexible membranes favor larger radii of curvature, while those with thicker, stiffer membranes adopt smaller sizes.

**5. Membrane permeability study**

**5a. Normalized data**


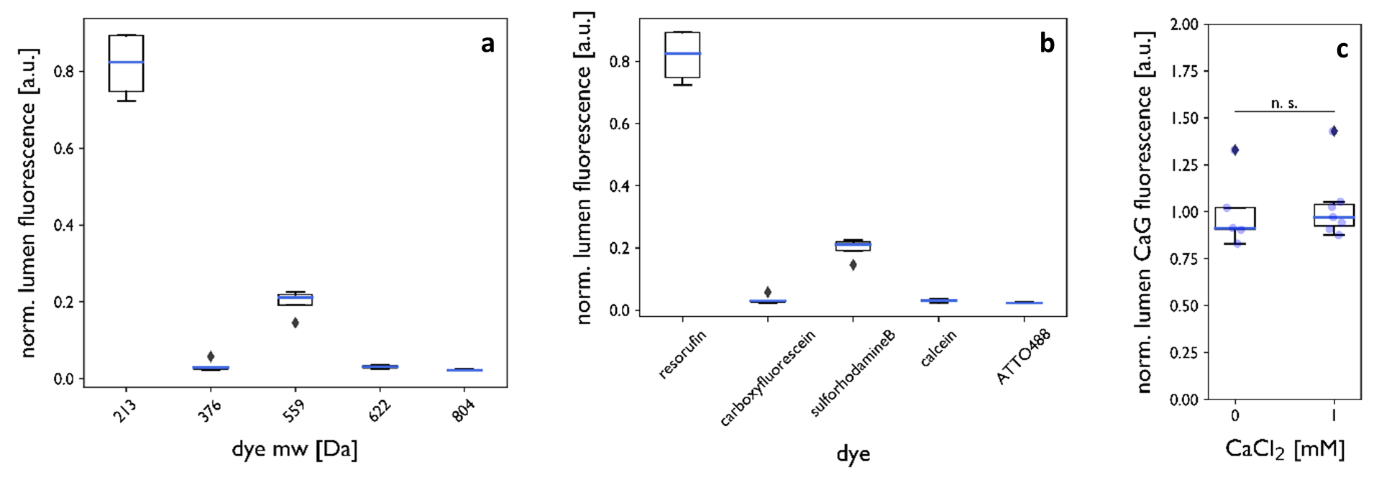


**Figure S14**. Membrane permeability analysis of GUVs (made from prop-PMOXA_10_-b-PDPA_16_) measured by normalizing the inner aqueous fluorescence after 2 h incubation with various fluorophores in the outer aqueous phase where the fluorophores are a) ordered by MW and b) listed by name.

**5b. Raw confocal laser scanning microscopy micrograph.**


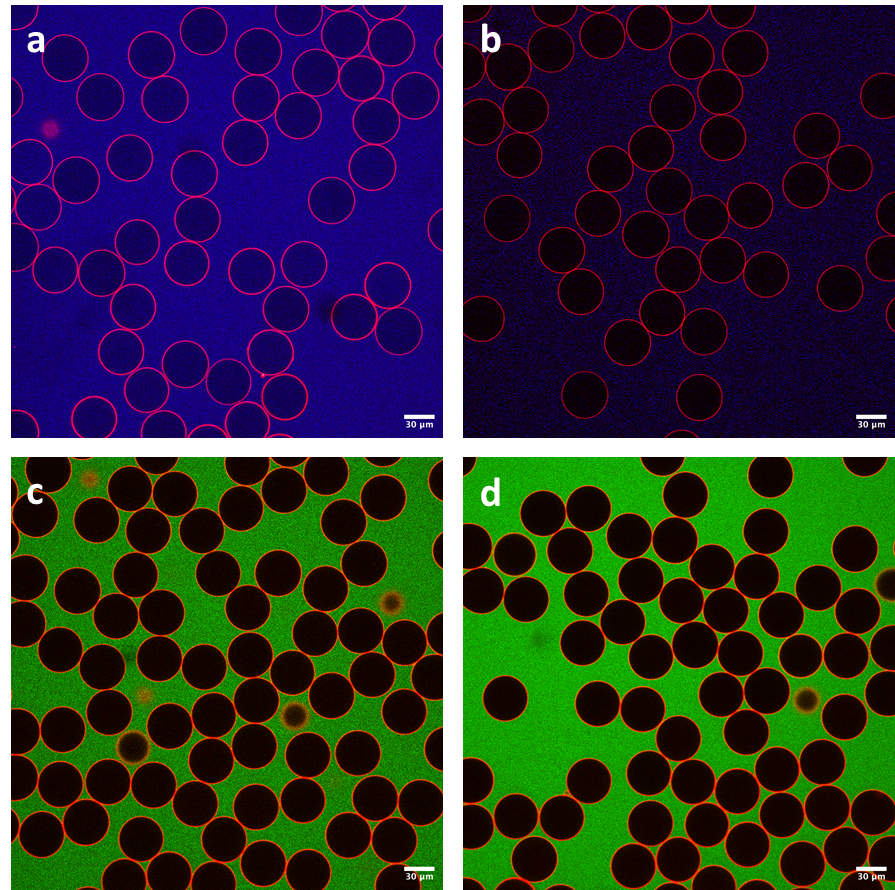


**Figure S15**. CLSM images used for fluorophore permeation statistics a) resorufin, b) sulforhodamine B, c) calcein and d) ATTO 488.

**6. Membrane Disruption**

**6a. FCS Measurements**

**Table S2.** Release of cargo from prop-PMOXA_10_-b-PDPA_16_ vesicles measured by FCS. Values are reported as SD, based on 20 independent measurements per sample.

| **Dye** | ***τ*_D Free Dye_**  **(µs)** | ***τ*_D_ _Loaded Dye_**  **(µs)** | ***Component Fraction _* *pH 5.6***  **(%)** | | **Calculated *R_h_^a^***  **(nm)** |
| --- | --- | --- | --- | --- | --- |
|  |  |  | ***Component 1*** | ***Component 2*** |  |
| **ATTO 488** | 39 ± 3.8 | 5956 ± 2419 | 98 ± 1 | 2 ± 1 | 94 ± 38 |
| **DOX∙HCl** | 30 ± 18 | 6726 ± 3455 | 88 ± 2 | 12 ± 2 | 137 ± 70 |

^a^R_h_ was determinated from Equation 5.

**Table S3.** Release of cargo from prop-PMOXA_21_-b-PDPA_34_ vesicles measured by FCS.

Values are reported as SD, based on 20 independent measurements per sample.

| **Dye** | ***τ*_D Free Dye_**  **(µs)** | ***τ*_D_ _Loaded Dye_**  **(µs)** | ***Component Fraction _* *pH 5.6***  **(%)** | | **Calculated *R_h_^a^***  **(nm)** |
| --- | --- | --- | --- | --- | --- |
|  |  |  | ***Component 1*** | ***Component 2*** |  |
| **ATTO 488** | 39 ± 3.8 | 3166 ± 1479 | 95 ± 2 | 5 ± 2 | 51 ± 24 |
| **DOX∙HCl** | 30 ± 18 | 4532 ± 1800 | 83 ± 3 | 17 ± 3 | 93 ± 37 |

^a^R_h_ was determinated from Equation 5.

**Table S4.** Membrane fragmentation of prop-PMOXa_10_-b-PDPA_16_ (**A_10_B_16_**) and prop-PMOXa_21_-b-PDPA_34_ (**A_21_B_34_**) vesicles stained with Nile Red and measured by FCS where component 1 = free dye, component 2 = membrane fragments and component 3 = vesicles. Values are reported as SD, based on 20 independent measurements per sample.

| **Polymer** | ***τ*_D_ _Vesicles_**  **(µs)** | ***Component Fraction (%)***  ***pH 5.6 - 5 mins*** | | | ***Component Fraction (%)***  ***pH 5.6 - 24 h*** | | | ***Component Fraction (%)***  ***pH 5.6 - 48 h*** | | | |
| --- | --- | --- | --- | --- | --- | --- | --- | --- | --- | --- | --- |
|  |  | ***1*** | ***2*** | ***3*** | ***1*** | ***2*** | ***3*** | ***1*** | ***2*** | ***3*** |  |
|  |  |  |  |  |  |  |  |  |  |  |  |
| **A_10_B_16_** | 13551 ± 2497 | 9 ± 2 | 22 ± 4 | 70 ± 3 | 41 ± 5 | 45 ± 6 | 14 ± 6 | 43 ± 6 | 44 ± 5 | 14 ± 9 |  |
| **A_21_B_34_** | 7807 ± 4171 | 0 | 38 ± 6 | 62 ± 4 | 9 ± 10 | 54 ± 4 | 37 ± 7 | 0 | 54 ± 8 | 46 ± 7 |  |

**6b. TEM Imaging**


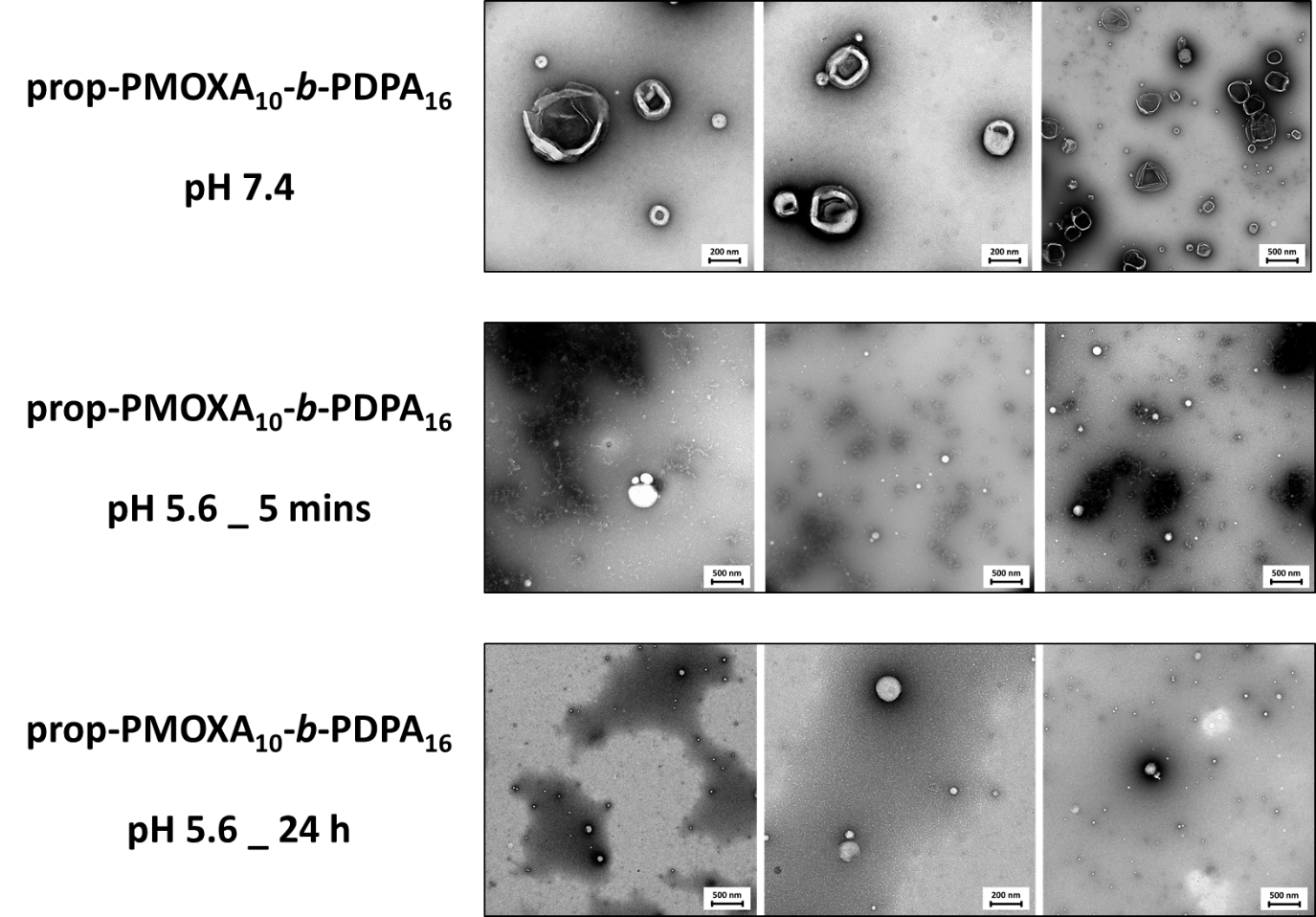


**Figure S16.** TEM micrographs of vesicles self-assembled from prop-PMOXA_10_-*b*-PDPA_16_ at pH 7.4 and showing different stages of membrane disruption at pH 5.6


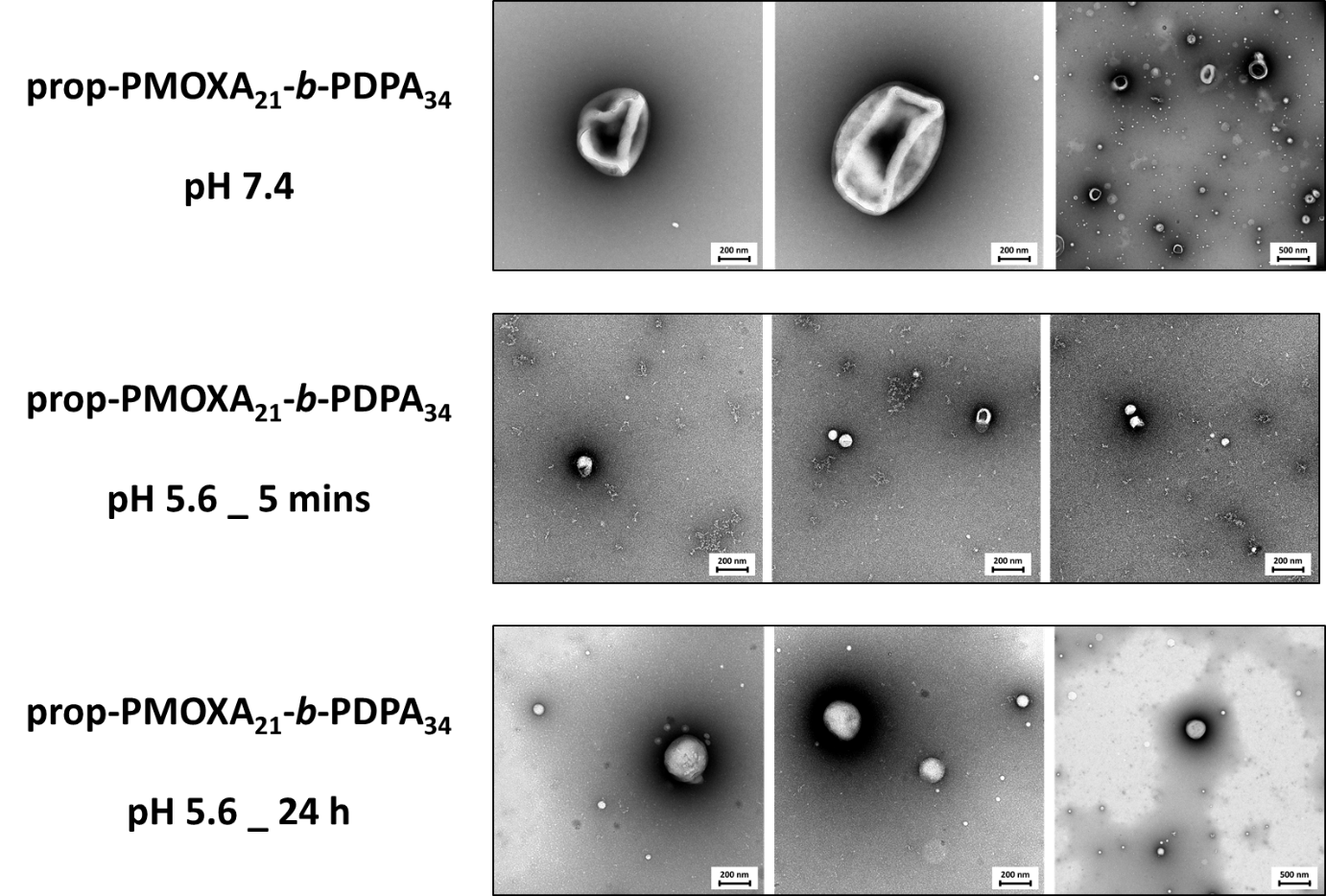


**Figure S17.** TEM micrographs of vesicles self-assembled from prop-PMOXA_21_-*b*-PDPA_34_ at pH 7.4 and showing different stages of membrane disruption at pH 5.6

**7. pH-Triggered Cargo Release**

**7a. Experiment Setup**

500 µL of each loaded vesicles (carboxyfluorescein, ATTO 488 and DOX∙HCl) were placed in dialysis bags (RC, MWCO = 8 kDa) and the bag was placed in 95 mL of PBS pre-adjusted to pH 5.6 (and pH 7.4 for the controls). Aliquots of 100 µL were taken from outside the dialysis bag at regular intervals and the samples were measured all measured using fluorimetry.

**7b. Exponential Decay Fitting Function**

All sample intensities were plotted against time and their maximum cumulative release was calculated according to the on-component exponential decay function (Equation SE1).

(SE1)

$$y= y_{max}+A_{1}e^{-x/t_{1}}$$

Where:

*y_max_* = maximum cumulative cargo release

*y* = cargo release

*x* = time

*A_1_* = scaling constant

*t_1_* = time constant

All fittings had an adjusted R^2^ value of at least 0.998.

**8. Cytotoxicity analysis**

**Figure S18**. Viability of HeLa cells after 48 h incubation with vesicle suspension at various concentrations and normalized according to the native cells used as control. Vesicle concentrations were derived from the polymer concentration used for self-assembly (5 mg mL^-1^).
